# Global stability of an age-structured population model on several temporally variable patches

**DOI:** 10.1007/s00285-021-01701-3

**Published:** 2021-12-04

**Authors:** Vladimir Kozlov, Sonja Radosavljevic, Vladimir Tkachev, Uno Wennergren

**Affiliations:** 1 Department of Mathematics, Linköping University, Linköping, Sweden; 2 Stockholm Resilience Centre, Stockholm University, Stockholm, Sweden; 3 Department of Physics, Chemistry, and Biology, Linköping University, Linköping, Sweden

**Keywords:** Age-structured population, Dispersal, Intra-cohort competition, Net reproductive number, Permanence, Strong monotonicity, 92D25, 35Q92, 35F31, 34D05

## Abstract

We consider an age-structured density-dependent population model on several temporally variable patches. There are two key assumptions on which we base model setup and analysis. First, intraspecific competition is limited to competition between individuals of the same age (pure intra-cohort competition) and it affects density-dependent mortality. Second, dispersal between patches ensures that each patch can be reached from every other patch, directly or through several intermediary patches, within individual reproductive age. Using strong monotonicity we prove existence and uniqueness of solution and analyze its large-time behavior in cases of constant, periodically variable and irregularly variable environment. In analogy to the next generation operator, we introduce the net reproductive operator and the basic reproduction number $R_0$ for time-independent and periodical models and establish the permanence dichotomy: if $R_0\le 1$, extinction on all patches is imminent, and if $R_0>1$, permanence on all patches is guaranteed. We show that a solution for the general time-dependent problem can be bounded by above and below by solutions to the associated periodic problems. Using two-side estimates, we establish uniform boundedness and uniform persistence of a solution for the general time-dependent problem and describe its asymptotic behaviour.

## Introduction

Population permanence in a patchy environment is a result of complex interactions between abiotic, biotic, and anthropogenic factors (Lewis et al. 2017). Each of these factors have relative importance for population growth with effects that may differ for terrestrial and aquatic species, large and small populations, plants and animals, vertebrates and invertebrates, Bjørnstad and Grenfell (2001), Kareiva and Wennergren (1995) and Roughgarden (1979). Understanding how interplay of spatial heterogeneity and temporal variability of the habitat, migration patterns, density-dependent feedback and age or class distribution of a population affect its dynamics and permanence is critical for science, conservation and management of biodiversity.

Mathematical models are often used for theoretical investigation of population dynamics and establishing conditions for population permanence. Despite spatial heterogeneity, temporal variability and variation in organization and functioning of natural ecosystems affected by human actions, population models typically emphasize importance of certain factors, while neglecting others. Some of the models highlight effects of age-structure and density- or time-dependence on population dynamics, Chipot (1983), Chipot (1984), Diekmann et al. (2001), von Foerster (1959), Gurtin and MacCamy (1974), Iannelli (1995), Iannelli and Pugliese (2014), Kozlov et al. (2016b, 2017) and Webb (1985). Iannelli and Milner (2017) presented a comprehensive study of age-structured population models and another significant contribution to this topic has recently been made by Inaba (2017). Age-structured models for two-sex populations have been studied in Iannelli et al. (2005). Some authors investigated *N*-species population with age-specific interactions and established the well-posedness and the existence of an equilibrium solution using the theory of semilinear evolution equations and derived local or asymptotic stability results, Prüß (1981) and Prüss (1983).

Spread of disease and epidemic modeling is an area where age-structured population dynamics plays a significant role. Recognition that transmission dynamics of some diseases cannot be explained by traditional models without age-structure sparked interest in analyzing the effects of age-structure on the dynamics of epidemics. These studies are sometimes limited to local results and investigating endemic equilibrium, such as Busenberg et al. (1988) and Castillo-Chavez et al. (1989), but there are also results related to global behavior of age-structured epidemic models, Busenberg et al. (1991), Feng et al. (2005), Kuniya and Iannelli (2014) and Kuniya et al. (2018).

A common feature of the above mentioned models is that they rarely include spatial heterogeneity as a factor of population growth. Models that do consider effects of habitat’s spatial structure on population permanence assume either discrete or continuous space. Discrete spatial structure means that a habitat consists of several distinct patches with birth and death rate being dependent on a patch that individuals occupy. A source is a high-quality patch that yields positive population growth, while a sink is a low-quality patch and it yields negative growth rate. In isolation, a subpopulation on every patch has its own dynamics. Linking patches by dispersal leads to the source-sink dynamics, where all local subpopulations contribute to a unique global dynamics. For populations that inhabit several patches, possibility to move from one patch to another can be crucial for survival. For example, dispersal from a source to a sink can save a local sink subpopulation from extinction through the rescue effect and recolonization (Amarasekare 2004; Dias 1996; Hastings 1993). The influence of spatial heterogeneity in unstructured populations is studied in Allen (1983), Arditi et al. (2015), Cui and Chen (1998), Cui and Chen (2001), DeAngelis et al. (2016), and DeAngelis and Zhang (2014). A trade-off between competition and dispersal is investigated in Amarasekare and Nisbet (2001) and the relation between dispersal pattern and permanence is discussed in Hastings and Botsfor (2006) and Jansen and Yoshimura (1998). Age-structured models on several patches usually assume discrete age classes (immature and adults) and dispersion between a few (two or three) temporally unchangeable patches, So et al. (2001), Takeuchi (1986a), Takeuchi (1986b), Terry (2011) and Weng et al. (2010). Age-structured population growth model in continuous environment typically represent movement as diffusion (Webb 2008).

On the mathematical side, these models have been studied using several main approaches. Integrated semigroup approach is one of them, see e.g. Magal and Ruan (2018), Magal et al. (2019) and Thieme (2009). The Lyapunov function approach has been used to establish (global) stability results in some epidemic models (Chekroun et al. 2019). A universal method for systematic construction of a Lyapunov function does not exists and this method is often of little use for complex dynamical systems; moreover, some Lyapunov functions may provide better answers than others. In contrast to this, monotonicity of certain positive operators and comparison principles have been used for studying global dynamics of ordinary, delay and partial differential equations, see Hirsch and Smith (2003), Smith (1995) and Zhao (2003). Application of these methods to age-structured population models and epidemic models can be found in Busenberg et al. (1991), Diekmann et al. (2001), Kuniya and Iannelli (2014), Kuniya et al. (2018), Magal and Ruan (2018), Magal et al. (2019) and Webb (1985).

In this paper we extend analysis to an age-structured population that inhabits *N* temporally variable patches connected by dispersal. Two key elements of our study are density-dependent population growth represented by pure intra-cohort competition (see also Kozlov et al. 2016a, 2017) and age- and time-dependent dispersal rates. From a biological point of view, intra-cohort competition occurs as a result of ageing and growth. Some species, such as certain insects, molluscs and fish, undergo metamorphosis, which is a complete change of physical appearance and structure and thereby also a change of food preferences and habitats. Individuals of different age therefore do not compete for resources, which reduces potential competitors from a whole population to a cohort. The pure intra-cohort competition is a relevant modeling approach when there are ontogenetic shifts. Age difference often correlates to body-size differences, which have effects on interactions between and within populations. Age difference is therefore viewed as a cause of diet and habitat shifts. Typically this is studied in food web theory, Petchey et al. (2008) and Zook et al. (2011), where diets are defined according to body-size, and in the theory of size-structured populations, Ebenman et al. (1996) and Narvaez et al. (2020). Pure intra-cohort competition is at one end of a continuum of how intraspecific competition may act and at the other end one finds the commonly used model of intraspecific competition that do not include structuring the population into, for example, age or body-size. In epidemiological modeling, intra-cohort transmission of a disease has been studied in Iannelli and Milner (2017, Section 1.3.5). The assumption that density-dependence has effects only on mortality rates is based on biological and modeling premises. Common individual responses to intraspecific competition and resource depletion are decreased fecundity and increased mortality. Effects of intraspecific competition on individuals may depend on individuals age, type of resource and type of competition (direct or indirect, interference or exploitation, see Gilad 2008). In some cases, lack of resources has stronger effect of mortality, while in other in affects fecundity more severely. From a modeling perspective high mortality rates of newborns can have the same effect as reduced fertility. To achieve nontrivial stability results and include these considerations, we assume that competition between individuals affects only mortality rates and that it occurs only between individuals of the same age (pure intra-cohort competition).

Some biological studies indicate that there are many different causes for dispersal, such as response to environmental conditions, prevention of inbreeding or competition for mates, and that migration can have different forms, such as one way dispersal or a round trip from a birthplace, Bowler and Benton (2005) and Dingle and Drake (2007). This may lead to differences in life-history traits, genetics and demography between dispersers and residents. Demographic studies show that dispersing females are often young individuals in their reproductive age, while old individuals usually do not engage in breeding dispersal, Gaines (1980) and Greenwood and Harvey (1982).

Taking into account these characteristics of density-dependent growth and migration, we formulate model as follow. Let $n_k(a,t)$ denote the age distribution in the population patch *k* at time *t* with the corresponding birth rate $m_k(a,t)$ and the initial distribution of population $f_k(a)$. A local subpopulation on each patch experiences intraspecific competition, which results in density-dependent mortality. The assumption that competition occurs only between members of the same age class leads to the following McKendrick–von Foerster type balance equations:

1 $$\begin{aligned} \frac{\partial {{\mathbf {n}}(a,t)}}{\partial t}+\frac{\partial {{\mathbf {n}}(a,t)}}{\partial a} = -{\mathbf {M}}({\mathbf {n}}(a,t),a,t){\mathbf {n}}(a,t)+{\mathbf {D}}(a,t){\mathbf {n}}(a,t) \end{aligned}$$

in the domain

2 $$\begin{aligned} {\mathscr {B}}:=\{(a,t)\in {\mathbb {R}}^{2}:0<a<B(t), \,\, t>0\} \end{aligned}$$

subject to the *birth law*

3 $$\begin{aligned} {\mathbf {n}}(0,t)=\int _0^{\infty }{\mathbf {m}}(a,t){\mathbf {n}}(a,t)\,da, \quad t> 0, \end{aligned}$$

and the *initial age distribution*

4 $$\begin{aligned} \mathbf{n }(a,0)=\mathbf{f }(a), \quad a>0. \end{aligned}$$

with

$$\begin{aligned} {\mathbf {n}}(a,t)&=(n_1(a,t), \ldots , n_N(a,t))^t,\\ {\mathbf {f}}(a,t)&=(f_1(a,t), \ldots , f_N(a,t))^t,\\ {\mathbf {m}}(a,t)&={{\,\mathrm{\mathrm {diag}}\,}}(m_1(a,t), \ldots , m_N(a,t)),\\ {\mathbf {M}}(n(a,t),a,t)&={{\,\mathrm{\mathrm {diag}}\,}}(M_1(n_1(a,t),a,t), \ldots , M_N(n_1(a,t),a,t)), \end{aligned}$$

where $M_k(v_k,a,t)$ is the density-dependent mortality rate of a population on patch *k* and dispersion matrix $\mathbf{D }(a,t)=(D_{kj}(a,t))_{1\le k,j\le N}$ defines migration rates between patches and describes migration pattern.

For ${\mathbf {D}}(a,t)\equiv 0$, there is no migration between patches and the system (1) splits into *N* independent balance equations. This model, under an additional assumption that ${\mathbf {M}}(a,t)$ is the logistic regulatory function (8), has recently been studied in Kozlov et al. (2017). The case ${\mathbf {D}}(a,t)\not \equiv 0$ is much more challenging. The coefficients $D_{kj}(a,t)$, $j\ne k$, define a proportion of individuals of age *a* at time *t* on patch *j* that migrates to patch *k*, which implies that $D_{kj}(a,t)\ge 0$ for all *a*, *t*. Given that migration is costly (in terms of time and energy) and risky for migrating individuals, we define $D_{kk}(a,t)$ as the migration-related mortality of individuals of age *a* at time *t* on patch *k* that is independent of the density-dependent mortality ${M_k}(n_k(a,t),a,t)$. The fact that individuals can disperse and move from one patch to another is important in modeling source-sink dynamics as it can explain effects of recolonization or population persistence in heterogeneous environment. It can be expected that the sign pattern and the weighted graph associated with $\mathbf{D }(a,t)$ affect global and asymptotic behaviour of solutions to (1)–(4).

In this paper, we provide a mathematical derivation of the results about the existence and uniqueness of a solution to the multi-patch model (1)–(4). The common point for the single-patch age-structured models is that *the basic reproduction number* $R_0$ and *the characteristic equation* are used to determine permanence of a population. The basic reproduction number represents expected number of offspring of an individual in constant environment. Its biological interpretation and computation in periodic environment have been discussed in Bacaër and Dads (2012) and Diekmann et al. (1990), respectively. In age-structured epidemic models, $R_0$ is the spectral radius of the next generation operator (Inaba 2019; Thieme 2003). The basic reproduction number is a threshold value that indicate long time behavior of a solution: if $R_0 < 1$, a population faces extinction, and if $R_0>1$, population persistence is granted, see Iannelli and Milner (2017), Inaba (2017, Section 1.4). Similar results hold for epidemic models, where the former condition means that the trivial disease-free equilibrium is globally asymptoticaly stable, while the latter condition implies global stability of a nontrivial equilibrium in which infected individuals persist (Chekroun et al. 2019). These results lead us to two key questions related to the multi-patch model (1)–(4):

- Is it possible to define an analogue of the characteristic equation and the basic reproduction number for the multi-patch model?
- If so, can they be used for the analysis of the large-time behavior of the solution and for establishing the condition for populations permanence?

The main contribution of the paper lies in a rigorous proof that both questions have affirmative answers in constant, periodic and general time-dependent cases. Similar result for time-independent case can be found in Thieme (2009, Section 6). A net reproductive dichotomy for an age-structured epidemic model in terms of disease persistence can be found in Thieme (2003, Sec.22.3). Our approach relies on the lower and upper solution technique and essentially uses monotonicity of certain integral operators associated with the balance equations. The method that we develop for the time-dependent cases allows investigation of asymptotic behaviour and global stability of the nonlinear model and considering fluctuations that are not necessarily small in amplitude. The obtained results enable discussion of conservation and management problems, such as improving survival of migrating species and pest control.

*Outline* A summary of the mathematical framework and our main results are presented in Sect. 2. In Sect. 3 we discuss an auxiliary model and derive some preliminary results on the corresponding lower and upper solutions. In Sect. 4 we prove the existence and uniqueness of a solution to the balance Eqs. (1)–(4) by reducing the original problem to a certain nonlinear integral equation. In Sect. 5 we define the associated characteristic equation and the maximal solution, and establish one of the key results of the paper: the basic reproduction number dichotomy. The remaining part of the paper is dedicated to the study of the asymptotic behavior and stability of the solution. We consider three cases: constant environment (i.e. the time-independent case) in Sect. 5, periodic environment in Sect. 6 and irregularly changing environment (i.e. the general time-dependent case) in Sect. 7.

*Notations* For easy reference we fix some standard notation used throughout the paper. ${\mathbb {R}}^{N}_+$ denotes the positive cone $\{x\in {\mathbb {R}}^N:x_i\ge 0\}$. Given $x,y\in {\mathbb {R}}^N$ we use the standard vector order relation: $x\le y$ if $x_i\le y_i$ for all $1\le i\le n$, $x< y$ if $x\le y$ and $x\ne y$, and $x\ll y$ if $x_i< y_i$ for all $1\le i\le n$. Given $x\in {\mathbb {R}}^{n}$,

$$\begin{aligned} \Vert x\Vert _{p}= \left\{ \begin{array}{ll} \left( \sum _{k=1}^N |x_k|^p\right) ^{1/p}, &{}\quad {1\le p<\infty ;} \\ \max _{1\le k\le N}|x_k|, &{}\quad {p=\infty .} \end{array} \right. \end{aligned}$$

In particular, if $D=D_{jk}$ is an $N\times N$-matrix we define $\Vert D_{jk}\Vert _p$ for any $1\le p\le \infty$ in an obvious manner identifying *D* with an element of ${\mathbb {R}}^{N^2}$. Given $E\subset {\mathbb {R}}^N$ and a continuous function $h:E\rightarrow {\mathbb {R}}$, we define

$$\begin{aligned} \Vert h\Vert _{C(E)}:=\sup _{x\in E}\Vert h_k(x)\Vert _\infty . \end{aligned}$$

## Main results

### The structure conditions

Before providing the main results, we give a brief summary of the structure conditions imposed on the balanced Eqs. (1)–(4). We always assume that ${\mathbf {m}}(a,t)$ and ${\mathbf {D}}(a,t)$ are continuous^1^ for $(a,t)\in {{\bar{{\mathscr {B}}}}}$ and ${\mathbf {M}}(v,a,t)$ is a continuous function of $(v,a,t)\in {\mathbb {R}}^{}\times {{\bar{{\mathscr {B}}}}}$. Following Iannelli (1995), we let $B(t)>0$ denote the *maximal length of life* of individuals in population at time $t\ge 0$. Then

$$\begin{aligned} {\mathbf {P}}(t)=\int _0^{B(t)}{\mathbf {n}}(a,t)\,da \end{aligned}$$

is the total population at time *t*. Furthermore suppose the following structure conditions hold:

1. there exists $0<b_1<b$ such that $b_1\le B(t)\le b$ for all $t\ge 0$ and
  5 $$\begin{aligned} \sup _{0<t_1<t_2<\infty }\frac{B(t_2)-B(t_1)}{t_2-t_1}<1 \end{aligned}$$
2. for any fixed $(a,t)\in {\mathscr {B}}$, $M_k(v,a,t)$ is a nonnegative nondecreasing function of *v* for $v\ge 0$, and there exist real numbers $\mu _\infty >0$, $\gamma >0$, and a function $p(a)\ge \mu _\infty$ such that
  6 $$\begin{aligned} M_k(v,a,t)-M_k(0,a,t)\ge p(a) v^{\gamma }, \quad \forall (v,a,t)\in {\mathbb {R}}^{}_+\times {\mathscr {B}}. \end{aligned}$$
3. $\Vert {\mathbf {D}}\Vert _{C({\mathscr {B}})}<\infty$ and ${\mathbf {D}}(a,t)$ is a *Metzler matrix*:
  7 $$\begin{aligned} D_{kj}(a,t)\ge 0, \qquad k\ne j; \end{aligned}$$
4. $\Vert {\mathbf {m}}\Vert _{C({\mathscr {B}})}<\infty$ and there exist $0<a_m<A_m<b_1$ such that
  $$\begin{aligned} {{\,\mathrm{\mathrm {supp}}\,}}{\mathbf {m}}\subset [a_m,A_m]\times {\mathbb {R}}^{+}. \end{aligned}$$
5. the function ${\mathbf {f}}(a)$ is continuous and ${{\,\mathrm{\mathrm {supp}}\,}}{\mathbf {f}}\subset [0,B(0))$.

Let us briefly explain the above conditions from the biological perspective. Concerning (H1), one usually uses a more restrictive condition that *B*(*t*) is a constant. Nevertheless, (5) is a more reasonable assumption: it means that the maximal length of life of individuals *B*(*t*) in a population may depend on *t* but it does not grow faster than time. Mathematically, (5) asserts that the boundary curve *B*(*t*) is transversal to the characteristics of (1).

The monotonicity assumption in (H2) ensures that increase in age-class density increases the death rate and has a negative effect on population growth. The classical example of the density independent mortality rate $M_k(v,a,t)=\mu _k(a,t)\ge \mu _\infty >0$ is compatible with $\gamma =0$ in (H2). Another example is the logistic type model (Kozlov et al. 2017) with

8 $$\begin{aligned} M_k(v,a,t)=\mu _k(a,t)\left( 1+\frac{v}{L_k(a,t)}\right) , \end{aligned}$$

where $L_k(a,t)\in L^\infty ({\mathscr {B}})$ is the regulatory function (carrying capacity); this example fits (H2) for $\gamma =1$.

Concerning the Metzler condition in (H3), note that the dispersion coefficient $D_{kj}(a,t)$ expresses the proportion of population $n_k(a,t)$ that from patch *j* goes to patch *k*, which naturally yields that $D_{kj}\ge 0$. Furthermore, according the support condition in (H4), the improper integral in (3) is well-defined and actually is taken over the finite interval $[a_m,A_m]$ which lies within the domain of definition of ${\mathbf {n}}(a,t)$ for any fixed $t>0$. The condition (H5) is a natural assumption that the initial distribution of population is bounded by the life length.

*The accessibility condition* For further applications we shall also need an additional assumption on the structure of the dispersion matrix ${\mathbf {D}}$. In order to formulate it, let us recall some relevant concepts. Given a Metzler matrix $A\in {\mathbb {R}}^{N\times N}$, one can associate a directed graph $\varGamma (A)$ with nodes labeled by $\{1,2,\ldots ,N\}$ where an arc leads from *i* to *j*, $i\ne j$, if and only if $A_{ij}> 0$. The patch *j* is said to be reachable from *i*, denoted $i\rightsquigarrow j$, if there exists a directed path from *i* to *j*. A digraph is called *connected from vertex* *i* if $i\rightsquigarrow j$ for all $j\ne i$ (Balakrishnan 1996, p. 132).

A patch *k* is said to be *accessible at age* $a\ge 0$ if the associated digraph $\varGamma (\mathbf{D }(a,t))$ is connected from *k* for any $t>0$. The accessibility condition relies on the sign pattern of the corresponding dispersion matrix and can be readily obtained by the standard tools of the nonnegative matrix theory (Minc 1988, Section 3).

Now, notice that by (H4) the following value is finite:

$$\begin{aligned} {{\bar{a}}}_k=\inf _{t>0}\sup \{ a: m_k(a,t)>0 \}\le A_m<\infty . \end{aligned}$$

From the biological point of view, ${{\bar{a}}}_k$ is the *maximal fertility age* in population *k*. Our last condition reads as follows:

(H6) For any $1\le k\le N$ there exists $0< \beta _k< {{\bar{a}}}_k$ such that the patch *k* is accessible at age $\beta _k$.

In other words, (H6) asserts that for any patch *k* there is a moment $\beta _k>0$ such that a (composite) migration from any other patch *j* to *k* is possible *within the reproductive period*. This condition is based on some biological studies showing that usually young individuals in their reproductive age engage in breeding dispersal, unlike old individuals who migrate less or migrate for other reasons.

### The net reproductive rate dichotomy

Let us denote by $\rho (t)=\mathbf{n }(0,t)$ the newborn function, i.e. a vector-function whose components denote the number of newborns on each patch. Then, the problem (1)–(4) can be reduced to the integral equation

9 $$\begin{aligned} \rho (t)={\mathcal {K}}\rho (t)+{\mathcal {F}}{\mathbf {f}}(t), \end{aligned}$$

where ${\mathcal {K}}$ and ${\mathcal {F}}$ are positive nondecreasing operators with bounded ranges and ${\mathcal {F}}{\mathbf {f}}(t)=0$ for large $t>0$. Our strategy for proving permanence results is as follows: we first establish the permanence results for time-independent and time-periodic coefficients, and then show that in the general situation, a solution of (9) can be well-controlled by these cases.

If the environment is constant then the model parameters are time-independent functions. Then it is reasonable to assume that the maximal life-time is constant: $B(t)\equiv b$ as in Chipot (1983) and Gurtin and MacCamy (1974). Our approach relies on a fine control of large-time behaviour of an arbitrary solution to (9) by nontrivial solutions of the associated *characteristic equation*

10 $$\begin{aligned} \rho =\bar{{\mathcal {K}}}\rho , \end{aligned}$$

where the operator $\bar{{\mathcal {K}}}$ is given by the right hand side in (3) for a time-independent solution to (1) with a constant boundary condition $\mathbf{n }(0)=\rho$. Clearly, $\rho =0$ is a (trivial) solution of the characteristic equation.

Our goal is to establish when a nontrivial positive solution $\rho \gg 0$ exists. A crucial tool here is the so-called *maximal solution* of the characteristic equation, i.e. a solution $\theta$ of (10) such that for an arbitrary solution $\rho$ there holds $\rho \le \theta$. In particular, $\theta =0$ implies that the characteristic equation has only trivial solutions. We establish the existence of the maximal solution in Sect. 5.2.

Another important ingredient is the next generation operator (see section 2.1 in Inaba 2017)

$$\begin{aligned} {\mathscr {R}}_0\rho =\int _0^{\infty }{\mathbf {m}}(a){\mathbf {Y}}(a;\rho )\,da, \end{aligned}$$

where ${\mathbf {Y}}(a;\rho )$ is the unique solution of the linearized initial problem

$$\begin{aligned} \frac{d {\mathbf {Y}}(a;\rho )}{da}=(-{\mathbf {M}}(0,a)+{\mathbf {D}}(a)){\mathbf {Y}}(a;\rho ), \quad {\mathbf {Y}}(0;\rho )=\rho \in {\mathbb {R}}^{N}_+. \end{aligned}$$

We show that under conditions (H1)–(H6), ${\mathscr {R}}_0:{\mathbb {R}}^{N}_+\rightarrow {\mathbb {R}}^{N}_+$ is a strongly positive operator. By Perron–Frobenius theorem, its spectral radius $R_0$ is equal to the largest positive eigenvalue. We call this value *the basic reproduction number*.

To motivate the latter definition, observe that in the single-patch case, the basic reproduction number $R_0$ is given by

$$\begin{aligned} R_0=\int _0^{\infty }m(a)e^{-\int _0^a\mu (v)dv}\,da. \end{aligned}$$

It it related to the solution of the Euler–Lotka characteristic equations in the linear age-structured population model; see Iannelli and Pugliese (2014). According to Kozlov et al. (2017), $R_0$ is related to the solution $\rho ^*$ of the characteristic equation in the nonlinear age-structured model. Namely, if $R_0\le 1$, then $\rho ^*=0$ and the population is going to extinction, while for $R_0>1$, we have $\rho ^*>0$ and the population is permanent. The same is obviously valid if there are several patches without migration (i.e. $\mathbf{D }\equiv 0$): every local subpopulation behaves accordingly to the value of $R_0$ on the respective patch.

The main contribution of this paper lies in the following dichotomy result on the long-term dynamics of populations.

#### Theorem B

(The Net Reproductive Rate Dichotomy) If $R_0\le 1$, then $\theta = 0$ and the characteristic Eq. (10) has no nontrivial solutions. If $R_0> 1$, then $\theta \gg 0$ and $\theta$ is the only nontrivial solution of the characteristic equation.

Let $\chi (t)$ be an arbitrary solution of (9). Then:

- If $R_0\le 1$, then $\chi (t)\rightarrow 0$ and ${\mathbf {P}}(t)\rightarrow 0$ as $t\rightarrow \infty$,
- If $R_0> 1$, then $\chi (t)\rightarrow \theta$ and ${\mathbf {P}}(t)\rightarrow \int _0^{\infty }\varphi (a;\theta )\,da$ as $t\rightarrow \infty$, where $\varphi (a;\theta )$ is the solution of the initial problem
  11 $$\begin{aligned} \frac{d}{da}\varphi (a;\theta )= -{\mathbf {M}}(\varphi (a;\theta ),a,t)\varphi (a;\theta )+ {\mathbf {D}}(a,t)\varphi (a;\theta ), \quad \varphi (0;\theta )=\theta .\nonumber \\ \end{aligned}$$

Thus, the basic reproduction number $R_0$ effectively determines large time behavior of population on *N* patches in a constant environment. Here, as in the single-patch case, $R_0\le 1$ implies extinction of a population on all patches, while $R_0> 1$ grants the global permanence of a population. We see that the dichotomy result for a multi-patch population is completely consistent with the single-patch case when the next generation operator ${\mathscr {R}}_0$ coincides with the multiplication by $R_0$.

It is important to emphasize that the function $\varphi (a;\theta )$ in (11) is exactly the unique equilibrium point of the problem (1), (3) provided that $\theta$ satisfies the characteristic equation. In other words, Theorem A implies the global stability result: any solution of the principal model converges at infinity to the unique equilibrium point given by the characteristic equation.

The proof of Theorem A, along with certain related results, occupies Sect. 5 and makes an essential use of the auxiliary monotonicity results collected in Sect. 3 and functional theoretic properties of the integral Eq. (9) given in Sect. 4. Our approach relies on the following steps and can be described as follows. First, we associate certain lower and upper monotone sequences to an arbitrary solution $\chi$ of (9). The existence of an upper sequence relies on the boundedness of the image of ${\mathcal {K}}$. The construction of a lower sequence is more tricky and involves certain fine properties of the maximal solution and some previous auxiliary monotonicity results together with the accessibility condition (H6). The main problem here is to control a nonzero asymptotic behaviour of the lower approximants as $t\rightarrow \infty$. Next, we show that the large-time behaviour of $\chi$ can be well controlled by the limits at infinity of constructed monotone approximants. Furthermore, we are able to identify the common limits as the maximal solution $\theta$. This finally establishes that the constructed sequences converge to the equilibrium point of the original problem. Notice that the monotonicity of the lower and upper approximations is crucial because the convergence established in the first steps is valid only on any bounded interval.

### Two-side estimates of $R_0$ and $\theta$

A life-history trade-off between reproduction and migration has been noted for many species, including migratory birds and some insects (see for example Guerra 2011; Mole and Zera 1993; Schmidt-Wellenburg et al. 2008). This trade-off is caused by energy constraints because both reproduction and migration are energetically costly for organisms. Keeping the assumption that the environment is constant and using the specific form of the balance system, we investigate the consequences of this trade-off.

The fact that individuals do not reproduce during migration is biologically justified and mathematically stated as:

12 $$\begin{aligned} \sum _{k=1}^ND_{kj}(a)\le 0, \quad 1\le j\le N. \end{aligned}$$

The relation (12) between dispersion coefficients represents total migration from patch *j* toward all other patches and means that some migrants that are leaving patch *j* will eventually die before reaching patch *k*, but they will not give birth during migration. This migration related mortality is represented by the nonpositive coefficient $D_{jj}(a)$, such that $|D_{jj}(a)|\ge \sum _{k=1, k\ne j}^ND_{kj}(a)$. Then, we establish in Sect. 5.6 the following two-side estimates for the basic reproduction number.

**Theorem B **Under additional assumption that (12) holds we have

$$\begin{aligned} \max _{1\le k\le N}\int _0^{\infty }m_k(a)e^{-\int _0^a(\mu _k(v)+|D_{kk}(v)|)dv}\,da&\le R_0\le \int _0^{\infty }m(a)e^{-\int _0^a\mu (v)dv}\,da, \end{aligned}$$

where *m*(*a*) is the maximal birth rate and $\mu (a)$ is the minimal death rate on all patches.

In addition, in Proposition 11 below we establish a priori estimates for the basic reproduction number and for the maximal solution $\theta$.

### Periodically and irregularly changed environment

Natural habitats are usually positively autocorrelated, see for example Steele (1985). Therefore, the assumption that the vital rates, regulating function and dispersal coefficients are changing periodically with respect to time is a reasonable approximation. In the study of the large-time behavior of a solution to Eq. (9) in a periodically changing environment, the pivotal role belongs to the characteristic equation

$$\begin{aligned} \rho (t)=\widetilde{{\mathcal {K}}}\rho (t), \end{aligned}$$

where the operator $\widetilde{{\mathcal {K}}}$ is given by the right hand side of (3) and ${\mathbf {n}}(a,t)$ solves (1) with a periodic boundary condition ${\mathbf {n}}(0,t)=\rho (t)$, $\rho (t+T)=\rho (t)$. We establish in Sect. 6 that the operator $\widetilde{{\mathcal {K}}}$ is absolutely continuous which allows us to extend the methods of Sect. 5 to the periodic case. In particular, the corresponding next generation operator $\widetilde{{\mathscr {R}}}_0$ defined on space of periodic continuous functions is strictly positive and its spectral radius ${R_0}$ is equal to the largest eigenvalue. We are also able to establish the corresponding dichotomy result for a periodic environment.

If the environment is changing irregularly, the structure parameters of the principal model (1)–(4) can be estimated from above and below by nonnegative periodic functions. Using these periodic functions as structure parameters for new models, we formulate two associated periodic problems. One of them is the best-case scenario and its solution is an upper bound for the original problem. The other is the worst-case scenario and its solution is a lower bound. In other words, a solution for the general time-dependent problem can be bounded for large values of *t* by above and below by the solution to the associated periodic problems, as stated in Theorem 7.

### Source-sink dynamics

Using the source-sink dynamics it is possible to explain permanence of a population on several patches provided that at least one patch is a source and that all patches are connected by dispersion. In Sect. 8.1 we assume that the environment is constant and consists of several patches. Then it is possible to show that survival of population on both patches is possible provided that emigration from the source is sufficiently small.

Furthermore, in Sect. 8.2, we show that permanence is possible even if all patches are sinks provided that dispersion is appropriately chosen. This is especially important for migratory birds, since both of their habitats can be seen as sinks (one because of the low reproduction due to insufficient resources, and the other because of the high mortality in the winter). This example can be related to the results in Jansen and Yoshimura (1998), where a simple model is used for analysis of connection between population permanence and allocation of offspring in a population that lives on several patches. One of the results is that permanence is possible even if all patches are sinks.

## An auxiliary model

### Upper and lower solutions

Below we establish some auxiliary monotonicity results for lower and upper solutions to a general system of ordinary differential equations

13 $$\begin{aligned} {\mathcal {L}}w:=\frac{d}{dx}w(x)-{\mathbf {F}}(w(x),x)=0, \quad x\in [0,b), \end{aligned}$$

where ${\mathbf {F}}(w,x):{\mathbb {R}}^{N}\times [0,b)\rightarrow {\mathbb {R}}^{N}$ is a locally Lipschitz function in $w\in {\mathbb {R}}^{N}$ for any $x\in [0,b)$ satisfying the *Kamke–Müller condition*, i.e. that the Jacobian matrix *DF*(*w*, *x*) is a Metzler matrix, i.e.

14 $$\begin{aligned} \frac{\partial F_i(w,x)}{\partial w_j}\ge 0 \quad i\ne j \end{aligned}$$

for almost all $w\in {\mathbb {R}}^{N}$ and all $x\in [0,b)$. We assume additionally that ${\mathbf {F}}$ satisfies

15 $$\begin{aligned} {\mathbf {F}}(0,x)= 0 \quad \text {for any}\;\, x\in [0,b). \end{aligned}$$

In particular, this implies that $w(x)\equiv 0$ is a solution of (13).

We shall also exploit a weaker version of the concept of irreducibility. More precisely, let $\mathbf{F }(w,x)=(F_1(w,x),\ldots , F_N(w,x) )$ be continuously differentiable with respect to *w* and let $D\mathbf{F }(w,x):=(\frac{\partial F_k(w,x)}{\partial w_j})$ denote the corresponding Jacobi matrix. Then an index $k\in \{1,2,\ldots , N\}$ is said to be $\mathbf{F }$-*accessible* at $x\in [0,b)$ if the associated digraph $\varGamma (D\mathbf{F }(w,x))$ is connected from *k* for any *w*.

In this paper, we are mostly interested in the particular case when

16 $$\begin{aligned} \mathbf{F }(w,x)=-\mathbf{M }({w},x){w}+ \mathbf{D }(x){w}, \quad x\in [0,b), w\in {\mathbb {R}}^{N}. \end{aligned}$$

then $D\mathbf{F }(w,x)=-{\mathbf {A}}+\mathbf{D }(x)$, where ${\mathbf {A}}=\mathrm {diag}[\partial _{w_i}(M_i(w_i,x)w_i)]$ is a diagonal matrix, and a patch $k\in \{1,2,\ldots , N\}$ is accessible at age *x* if $\varGamma (\mathbf{D }(x))$ is connected from *k*. Note also that if ${\mathbf {F}}$ is defined by (16) then (14) is equivalent to that $D{\mathbf {F}}(w,x)=-{\mathbf {A}}+{\mathbf {D}}(x)$ is a Metzler matrix. In this case the condition (15) is trivially satisfied.

#### Definition 1

A locally Lipschitz function *w*(*x*) is called an *upper* (resp. *lower*) solution to (13) if $\frac{d}{dx}w(x)\ge {\mathbf {F}}(w(x),x)$ (resp. $\frac{d}{dx}w(x)\le {\mathbf {F}}(w(x),x)$) holds for all $x\in [0,b)$.

The next lemmas generalize the corresponding facts for the cooperative system (cf. Smith 1995, Remark 1.2) on lower (upper) solutions of (13) with Lipschitzian ${\mathbf {F}}$. Notice also that our proofs are somewhat different from those given in Smith (1995). Let us agree to write

$$\begin{aligned} v\ge _{k} u \quad \Leftrightarrow \quad v\ge u \quad \text {and}\quad v_k=u_k\quad \text {for some}\;\,1\le k\le N. \end{aligned}$$

First notice that ${\mathbf {F}}$ satisfies the so-called quasimonotone condition (Hirsch and Smith 2003; Smith 1995).

#### Lemma 1

If ${\mathbf {F}}$ satisfies the Kamke–Müller condition then $u\le _{k} v$ implies $F_k(u,x)\le F_k(v,x)$ for any $x\in [0,b)$.

#### Proof

Indeed, the function $g(t)={\mathbf {F}}(u+t(v-u),x)$ is absolutely continuous in [0, 1], hence applying by the fundamental theorem of calculus and (14) that

17 $$\begin{aligned} \begin{aligned} F_k(v,x)-F_k(u,x)&=\int _{0}^1g'_k(t)dt\\&=\int _{0}^1 \sum _{i=1}^N\frac{\partial F_k(u+t(v-u),x)}{\partial w_i}(v_i-u_i) dt\\&=\sum _{i=1,i\ne k}^N(v_i-u_i)\int _{0}^1\frac{\partial F_k(u+t(v-u),x)}{\partial w_i} dt\ge 0,\\ \end{aligned} \end{aligned}$$

as desired. $$\square $$

#### Lemma 2

Let *w*(*x*) be an upper solution of (13) a.e. in [0, *b*) such that $w(0) \ge 0$. Then $w(x)\ge 0$ on [0, *b*). Furthermore, if $w_j(0)>0$ then $w_j(x) > 0$ for $x\in [0,b)$.

#### Proof

First we claim that $w(x)^-:=(w_1^-(x),\ldots ,w_N^-(x))$ is also an upper solution of (13) a.e. in [0, *b*), where $w_k^-(x)=\min (0,w_k(x))$. Indeed, since each $w^-_k(x)$ is a locally Lipschitz function, there exists a full Lebesgue measure subset $E\subset (0,b)$ where all $w^-_k(x)$ are differentiable. We will show that $w^-$ satisfy $(w^-)'(x)\ge {\mathbf {F}}(w^-(x),x)$ on *E*. Let $x_0\in E$ and $1\le k\le N$. If $w_k(x_0)\ge 0$ for some *k* then $w_k^-(x_0)=0$, hence $x_0$ is a local maximum of $w_k^-(x)$ (because $w_k^-(x)\le 0$ everywhere). This yields $(w_k^-)'(x_0)=0$. Furthermore, since $0\ge _{k} w_-(x_0)$, we have by Lemma 1 and (15) that

$$\begin{aligned} (w_k^-)'(x_0)=0=F_k(0,x_0)\ge F_k(w^-(x_0),x_0). \end{aligned}$$

If $w_k(x_0)< 0$ then by the continuity of $w_k(x)$ one has $w_k^-(x)=w_k(x)$, $(w_k^-)'(x)=w_k'(x)$ in some neighbourhood of $x_0$. Thus, applying (13) we have by $w(x)\ge _{k}w^-(x)$ and Lemma 1 that

$$\begin{aligned} (w_k^-)'(x)=w_k'(x) \ge F_k(w(x),x) \ge F_k(w^-(x),x) \end{aligned}$$

holds everywhere in the neighbourhood of $x_0$. Thus, the claim is proved.

We also claim that any upper solution to (13) with $w(0)= 0$ and $w(x)\le 0$ for $x\in [0,b)$ is identically zero in the interval. Indeed, if *w* is such a function then let *T* be chosen as the supremum of all $t\in [0,b)$ such that $w(x)=0$ in [0, *t*]. If $T=b$ the claim is proved. Therefore assume that $T<b$. Then by the continuity $w(T)=0$ and for any $\epsilon >0$ there exists $x\in [T,T+\epsilon ]$ such that $w(x)<0$, and thus $\Vert w(x)\Vert _1>0$. Since ${\mathbf {F}}(w,x)$ is locally Lipschitz in *w*, there exist $M>0$ and $\epsilon >0$ such that $\Vert {\mathbf {F}}(w,x)-{\mathbf {F}}(0,x)\Vert _{1}\le M\Vert w\Vert _{1}$ for any $\Vert w\Vert _1<\epsilon$ and any $x\in [0,b)$. Define $h(x)=\Vert w(x)\Vert _1\equiv -\sum _{i=1}^N w_i(x)$ (recall that by the assumption $w_i(x)\le 0$ for all *i* and $x\in [0,b)$). By the continuity of *w*(*x*), there exists $\delta$ such that $\Vert w(x)\Vert _1<\epsilon$ for any $|x-T|<\delta$. Let the set *E* be defined as above and $x\in [T,T+\delta )$. Since by (15) ${\mathbf {F}}(0,x)=0$, we have

$$\begin{aligned} h'(x)=-\sum _{i=1}^N w'_i(x)\le -\sum _{i=1}^N F_i(w(x),x)\le M\Vert w(x)\Vert _1=Mh(x). \end{aligned}$$

The latter inequality yields $(h(x)e^{-Mx})'\le 0$ a.e. in $[T,T+\delta ]$. Since *h*(*x*) is locally Lipschitz it is absolutely continuous, thus $h(x)e^{-C(a)x}\le h(T)=0$ in $[T,T+\delta ]$, i.e. $\Vert w(x)\Vert _1\equiv 0$ in the interval, a contradiction with the choice of *T*. This yields the claim.

Now, if *w*(*x*) is an upper solution to (13) with $w(x)\ge 0$ then by the first claim $w^-(x)$ is an upper solution solution with $w^{-}(0)=0$. Then the second claim implies $w^-(x)\equiv 0$ in [0, *b*), thus we have $w(x)\ge 0$ in [0, *b*).

To finish the proof, let us suppose that $w_j(0)>0$. Since $F_j(y,x)$ is locally Lipschitz in *y*, for any $r>0$ there exists *C*(*r*) such that [in virtue of (15)] $|F_j(y,x)|\le C(r)\Vert y\Vert _1$ for all $y\in {\mathbb {R}}^{N}$ and $\Vert y\Vert \le r$. Let $0<\beta <b$ be chosen arbitrarily and let $r=\sup _{x\in [0,\beta ]}|w_j(x)|$. Since $w(x)\ge _j w_j(x)e_j$, where $e_j$ is the *j*th coordinate vector, Lemma 1 and the nonnegativity of $w_j(x)$ yield that

$$\begin{aligned} \frac{d}{dx}w_j(x)\ge F_j(w(x),x)\ge F_j(w_j(x)e_j,x)\ge -C(r)w_j(x), \quad x\in [0,\beta ]. \end{aligned}$$

The latter yields $w_j(x)e^{C(r)x} \ge w_j(0)>0$, thus $w_j(x)>0$ for every $x\in [0,\beta ]$, and therefore in the whole interval [0, *b*). $\square$

#### Lemma 3

Let *w*(*x*) be an upper solution of (13) with $w(0)> 0$ and such that the *k*-th patch is ${\mathbf {F}}$-accessible at some $\beta \in [0,b)$ then $w_k(x) > 0$ on $(\beta ,b)$.

#### Proof

It follows from Lemma 2 that if $w_k(\beta )>0$ then $w_k(x)>0$ holds everywhere in $[\beta ,b)$. Therefore we may without loss of generality assume that $w_k(\beta )=0$. Let us suppose by contradiction that there exists $\beta _1\in (\beta ,b)$ such that $w_k(\beta _1)=0$. Then $w_k(x)\equiv 0$ in $[0,\beta _1]$. In particular, $w_k'(\beta )=0$. Since $w(0)>0$, there exists *j* such that $w_j(0)>0$ and, thus, $w_j(\beta )>0$. By the assumption, there exists a directed path $k\rightsquigarrow j$ in the graph $\varGamma (D{\mathbf {F}}(w,\beta ))$. Equivalently, there exists a sequence of pair-wise distinct $j_0=k$, $j_1,\ldots ,j_{s-1}$, $j_s=j$ such that

18 $$\begin{aligned} \frac{\partial F_{j_{i}}}{\partial w_{j_{i+1}}}(w(\beta ),\beta )>0,\quad \forall i=0,1,\ldots , s-1. \end{aligned}$$

For any $i=0,\ldots , s-1$, let us define

$$\begin{aligned} v_0:=w(\beta ), \quad v_i:=w(\beta )-(w_{j_1}(\beta )e_{j_1}+\ldots +w_{j_i}(\beta )e_{j_i}),\quad 1\le i\le s, \end{aligned}$$

where $e_i$ denotes the *i*th coordinate unit vector in ${\mathbb {R}}^{N}$. Then

19 $$\begin{aligned} w(\beta )= v_0\ge _{j_0} v_1\ge _{j_1} \ldots \ge _{j_{s-1}} v_{j_s}=v_{j}\ge 0. \end{aligned}$$

Since *w*(*x*) is an upper solution of (13), we have by Lemma 1 for $j_0=k$ that

$$\begin{aligned} 0=w_{j_0}'(\beta )\ge F_{j_0}(v_0,\beta )\ge F_{j_0}(v_1,\beta )\ge F_{j_0}(0,\beta )=0, \end{aligned}$$

hence $F_{j_0}(v_0,\beta )= F_{j_0}(v_1,\beta )=0$. Arguing as in (17) we find

20 $$\begin{aligned} \begin{aligned} 0&=F_{j_0}(v_0,\beta )-F_{j_0}(v_1,\beta )\\&=\sum _{i=1,i\ne j_0}^N(v_0-v_1)_i\int _{0}^1\frac{\partial F_{j_0}(v_0+t(v_0-v_1),\beta )}{\partial w_i} dt\\&\ge 0. \end{aligned} \end{aligned}$$

It follows from (20), the nonnegativity of $(v_0-v_1)_{i}$ and the partial derivatives (for $i\ne j_0$) that all summands of the latter sum must vanish. Since the integrands are non-negative continuous functions, they must vanish identically for $t\in [0,1]$. In particular, (18) readily implies that $(v_0-v_1)_{j_1}=0$. Thus, $w_{j_1}(\beta )=0$, and by the above we have $w'_{j_1}(\beta )=0$

Repeating the same argument for the pair $(j_1,j_2)$ etc. implies $w_{j_2}(\beta )=0$ etc., thus yielding that $w_{j_s}(\beta )=w_{j}(\beta )=0$, a contradiction follows. $\square$

#### Proposition 1

(Comparison principle) Let *u*(*x*) and *v*(*x*) be resp. upper and lower solutions to (13) such that $u(0) \ge v(0)$. Then $u(x)\ge v(x)$ for all $x\in [0,b)$. If additionally the patch *k* is ${\mathbf {F}}$-accessible at some $\beta \in [0,b)$ and $u(0) > v(0)$ then $u_k(x)> v_k(x)$ for all $x\in (\beta ,b)$. If particular, if (13) is irreducible and $u(0) > v(0)$ then $u(x)\gg v(x)$ for all $x\in (0,b)$.

#### Proof

Let $$w(x)=u(x)-v(x)$$. Then

$$\begin{aligned} w'(x)\ge {\mathbf {F}}(v(x)+w(x),x)-{\mathbf {F}}(v(x),x)={\mathbf {G}}(w(x),x), \end{aligned}$$

i.e. *w*(*x*) is an upper solution to ${\mathcal {L}}_G w:=\frac{d}{dx}w(x)-G(w(x),x)$ with $G(\xi ,x):={\mathbf {F}}(v(x)+\xi ,x)-{\mathbf {F}}(v(x),x)$. We have for the corresponding Jacobi matrices

$$\begin{aligned} DG(\xi ,x)=D {\mathbf {F}}(\xi +v(x),x), \end{aligned}$$

i.e. ${\mathcal {L}}$ and ${\mathcal {L}}_g$ satisfy simultaneously the Kamke–Müller condition. This readily yields the first claim of the proposition.

Now let us assume that $u(0) > v(0)$ and for some *k* and $\beta \in [0,b)$ the associated digraph $\varGamma (D{\mathbf {F}}(w(\beta ),\beta ))$ is connected from *k*. Since $D{\mathbf {G}}(w(\beta ),\beta )=D{\mathbf {F}}(u(\beta ),\beta )$ the digraph $\varGamma (D{\mathbf {G}}(w(\beta ),\beta ))$ is also connected from *k*. Applying Lemma 3 we deduce $w_k(x)>0$, i.e. $u_k(x)> v_k(x)$ for all $x\in (\beta ,b)$, as desired. $\square$

#### Corollary 1

Let *u*(*x*) be an lower (resp. upper) solution to (13). If $u(0)\le 0$ (resp $u(0)\ge 0$) then $u(x)\le 0$ (resp. $u(x)\ge 0$ ) for all $x\in [0,b)$. If additionally the patch *k* is ${\mathbf {F}}$-accessible at some $\beta \in [0,b)$ and $u(0) < 0$ (resp. $u(0)>0$) then $u_k(x)< 0$ (resp. $u_k(x)> 0$) for all $x\in (\beta ,b)$.

#### Proof

Follows immediately from the fact that $w(x)\equiv 0$ is a solution of (13). $\square$

#### Proposition 2

(Existence and Uniqueness) Let (13) satisfy the Kamke–Müller condition and there exists $C({\mathbf {F}})>0$ such that

21 $$\begin{aligned} \max _{k}F_k(w,x)\le C({\mathbf {F}})\Vert w\Vert _\infty , \quad \forall w\in {\mathbb {R}}^{N}_+,\, x\in [0,b). \end{aligned}$$

Then for any $\xi \in {\mathbb {R}}^{N}_+$ there exists a unique solution $w(x)\in C^1([0,b),{\mathbb {R}}^{N}_+)$ of (13) with $w(0)=\xi$. Furthermore, if *w*(*x*) is a nonnegative lower solution to (13) then

22 $$\begin{aligned} \Vert w(x)\Vert _\infty \le \Vert w(0)\Vert _\infty e^{C({\mathbf {F}})b}. \end{aligned}$$

#### Proof

By the Cauchy–Peano Existence Theorem, (13) has a unique solution *w*(*x*) in some interval $$[0,\beta )$$, $$0<\beta \le b$$. By Lemma 2, $$w(x)\ge 0$$ for any $$x\ge 0$$ in the domain of the definition. Let $$[0,b')$$ be the maximal interval of existence of the solution:

$$\begin{aligned} b':=\sup \{\beta >0: \text { there exists a solution of (13) on }[0,\beta ) \}. \end{aligned}$$

We claim that $b' =b$. It suffices to show that a solution *w*(*x*) is uniformly bounded on any existence interval $[0,\beta )$, i.e. there exists $M>0$ such that for any $\beta <b'$ the inequality $\Vert w(x)\Vert _{\infty }\le M$ holds in $[0,\beta )$. To this end, we make a more general assumption, that *w*(*x*) is a *nonnegative* lower solution to (13) on $[0,\beta )$ and consider

$$\begin{aligned} H(x)=\Vert w(x)\Vert _{\infty }=\max _{k}w_k(x). \end{aligned}$$

In particular, *H*(*x*) is locally Lipschitz on $[0,\beta )$, and thus a.e. differentiable there. Then for any point of differentiability *x* of *H* there exists *k* such that $H(x)=w_k(x)$ and $H'(x)=w'_k(x)$. We have $w(x)\le _k H(x){\mathbf {1}}$ which implies by Lemma 1 and (21) that

23 $$\begin{aligned} H'(x)=w'_k(x)\le F_k(w(x),x)\le F_k(H(x){\mathbf {1}},x)\le C({\mathbf {F}})H(x). \end{aligned}$$

Integrating the latter inequality (note that *H* is absolutely continuous) yields

$$\begin{aligned} H(x)\le H(0)e^{C({\mathbf {F}})x}\le \Vert w(0)\Vert _\infty e^{NC({\mathbf {F}})b}. \end{aligned}$$

This proves (22). Furthermore, since the latter upper bound is independent of $\beta$, this implies $b'=b$, and thus the existence and the uniqueness of solution of (13) on [0, *b*). $\square$

### Further estimates for concave ${\mathbf {F}}$

To proceed we consider some additional assumptions on ${\mathbf {F}}$. Namely, a vector-function $F\in C({\mathbb {R}}^{N},{\mathbb {R}}^{N})$ is said to be *concave* if

24 $$\begin{aligned} {\mathbf {F}}(\alpha _1 u+\alpha _2 v)\le \alpha _1 {\mathbf {F}}(u)+\alpha _2 {\mathbf {F}}(v), \quad \forall \alpha _i\ge 1, \,\,u,v\in {\mathbb {R}}^{N}. \end{aligned}$$

A concave vector-function ${\mathbf {F}}$ is said to be *strongly concave* if for any $\alpha >1$ and any $u\ge 0$ with $u_k>0$ there holds

25 $$\begin{aligned} F_k(\alpha u)< \alpha F_k(u). \end{aligned}$$

#### Corollary 2

Let ${\mathbf {F}}$ be a concave vector-function satisfying the Kamke–Müller condition. Let *v*(*x*) be a lower and *u*(*x*) be an upper solutions of (13). Then $v(x)-u(x)$ is a lower solution of (13).

#### Proof

The claim follows from (24) with $\alpha _1=\alpha _2=1$:

$$\begin{aligned} v'(x)-u'(x)\le {\mathbf {F}}(v(x),x)-{\mathbf {F}}(u(x),x)\le {\mathbf {F}}(v(x)-u(x),x). \end{aligned}$$

$$\square $$

#### Corollary 3

Let ${\mathbf {F}}$ be a concave vector-function satisfying the Kamke–Müller condition and (21). If *v*(*x*), *u*(*x*) are solutions of (13) with $v(0)\ge 0,u(0)\ge 0$ then

26 $$\begin{aligned} \Vert v(x)- u(x)\Vert _{C[0,b)} \le e^{C({\mathbf {F}})b}\Vert v(0)-u(0)\Vert _{\infty }. \end{aligned}$$

#### Proof

By the assumptions $u(0),v(0)\in {\mathbb {R}}^{N}_+$. First suppose that $v(0)\ge u(0)$ and define $w(x)=v(x)-u(x)$. Then by Proposition 1, $w(x)\ge 0$ for any $x\in [0,b)$. Therefore by Corollary 2*w* is a (nonnegative) lower solution to (13), thus by Proposition 2 we have $\Vert w(x)\Vert _{C[0,b)} \le e^{C({\mathbf {F}})b}\Vert w(0)\Vert _{\infty },$ which proves (26).

In the general case, let *w*(*x*) be the solution of (13) with the initial conditions $w_k(0)=\min (u_k(0),v_k(0))$, $1\le k\le N$. Then $u(0)\ge w(0)$ and $v(0)\ge w(0)$, hence by the above

27 $$\begin{aligned} \begin{aligned} \Vert u(x)- w(x)\Vert _{C[0,b)}&\le e^{C({\mathbf {F}})b}\Vert u(0)- w(0)\Vert _{\infty }\\ \Vert v(x)- w(x)\Vert _{C[0,b)}&\le e^{C({\mathbf {F}})b}\Vert v(0)- w(0)\Vert _{\infty }. \end{aligned} \end{aligned}$$

Since $u(x)\ge w(x)$ and $v(x)\ge w(x)$ for any $x\in [0,b)$ we also have

$$\begin{aligned} w(x)-v(x)\le u(x)- v(x)\le u(x)-w(x), \end{aligned}$$

which by virtue of (27) yields

$$\begin{aligned} \Vert u(x)- v(x)\Vert _{C[0,b)}\le e^{C({\mathbf {F}})b}\max \{\Vert u(0)- w(0)\Vert _{\infty },\Vert v(0)- w(0)\Vert _{\infty }\} \end{aligned}$$

On the other hand, by our choice, for any *k* there holds that

$$\begin{aligned} \max \{|u_k(0)-w_k(0)|,|v_k(0)-w_k(0)|\}=|u_k(0)-v_k(0)|, \end{aligned}$$

hence

$$\begin{aligned} \max \{\Vert u(0)- w(0)\Vert _{\infty }, \Vert v(0)- w(0)\Vert _{\infty }\}\le \Vert u(0)-v(0)\Vert _\infty . \end{aligned}$$

which yields (26). $$\square $$

#### Proposition 3

Let $\phi (x,\xi )$ denote the solution *w*(*x*) of problem (13) in [0, *b*) with the initial condition $w(0)=\xi \in {\mathbb {R}}^{N}_+$. Suppose ${\mathbf {F}}$ satisfy the Kamke–Müller condition and that it is concave. Then

28 $$\begin{aligned} \phi (x,\alpha \xi )\le \alpha \phi (x,\xi ), \quad \forall \alpha \ge 1, \forall x\in [0,b). \end{aligned}$$

Let additionally ${\mathbf {F}}(w,x)$ be strongly concave, $\xi > 0$ and $\alpha >1$. If the patch *k* is ${\mathbf {F}}$-accessible at some $\beta \in [0,b)$ then

29 $$\begin{aligned} \phi _k(x,\alpha \xi )< \alpha \phi _k(x,\xi ), \quad \forall x\in (\beta ,b). \end{aligned}$$

#### Proof

Define $u(x)= \phi (x,\xi )$, $v(x)= \phi (x,\alpha \xi )$ and $w(x)=\alpha \phi (x,\xi )$. By the concavity condition,

30 $$\begin{aligned} {\mathcal {L}}w={\mathcal {L}}(\alpha u)\ge \alpha {\mathcal {L}}(u)\ge 0 \quad \forall x\in [0,b), \end{aligned}$$

where ${\mathcal {L}}u=\frac{du}{dx}-{\mathbf {F}}(u(x),x)$. In other words, *w*(*x*) is an upper solution with

$$\begin{aligned} w(0)=v(0)=\alpha \xi , \end{aligned}$$

hence Proposition 1 yields $w(x)\ge v(x)$ for $x\in [0,b)$. This yields (28).

Now, suppose that ${\mathbf {F}}(w,x)$ is strongly concave, $\xi > 0$, $\alpha >1$ and patch *k* is ${\mathbf {F}}$-accessible at some $\beta \in [0,b)$. By virtue of (28), it suffices to show that the equality $w_k(x)=v_k(x)$ is impossible in $(\beta ,b)$. Arguing by contradiction let us assume that there exists $x_0\in (\beta ,b)$ such that $w_k(x_0)=v_k(x_0)$. We claim that in this case $w_k(x)\equiv v_k(x)$ for any $x\in [\beta ,x_0)$. Indeed, if not then there exists $x_1\in (\beta ,x_0)$ such that $w_k(x_1)>v_k(x_1)$, hence the second part of Proposition 1 implies $w_k(x)> v_k(x)$ for any $x\in (x_1,b)$, a contradiction at the point $x_0$ follows. Thus, $w_k(x)\equiv v_k(x)$ and, thus,

31 $$\begin{aligned} {\mathcal {L}}w(x)=0\quad \text {for any}\quad x\in [\beta ,x_0). \end{aligned}$$

On the other hand, by the assumption $u(0)=\xi > 0$ and Corollary 1 we have $u_k(x)>0$ for $x\in (\beta ,b)$. Using the strong concavity condition (25), $F_k(\alpha u(x),x)< \alpha F_k(u(x),x)$ for $x\in (\beta ,b)$ which yields ${\mathcal {L}}w(x)=({\mathcal {L}}(\alpha u))(x)> \alpha {\mathcal {L}}(u(x))=0$, a contradiction with (31) completes the proof. $\square$

## The main representation

We start with an auxiliary model (36) below and then prove the existence of a unique positive solution of (1)–(4) and examine asymptotic behavior of the obtained solution. Everywhere in this section we assume the conditions (H1)–(H4) are satisfied.

### The balanced equations

Now we consider the particular case of (13) with ${\mathbf {F}}(w,x)$ given by (16). In other words, we consider the differential operator

32 $$\begin{aligned} {\mathcal {L}}w(x)=\frac{d w(x)}{dx}+{\mathbf {M}}(w(x),x)w(x)- {\mathbf {D}}(x)w(x). \end{aligned}$$

For further applications, it is useful to specify the properties of $M_k$. Recall that in the Lotka–McKendrick–von Foester model (1) with (8) each $M_k(v,x)$ is actually an *increasing linear* function in *v*. While keeping monotonicity, we also impose some additional growth conditions on $M_k$. Namely, we suppose that each $M_k(v,x)$ satisfies (H2), i.e. it is a nonnegative continuous function on ${\mathbb {R}}^{}\times [0,b)$,

33 $$\begin{aligned} M_k(v,x) \text { is strongly increasing in}\; v\ge 0\; \text {for any fixed}\; x\in [0,b) \end{aligned}$$

and there exist $\gamma >0$ and $\mu _\infty >0$ such that

34 $$\begin{aligned} M_k(v,x)-\mu _k(x)\ge \mu _\infty v^{\gamma }, \quad \forall (v,x)\in {\mathbb {R}}^{}_+\times [0,b), \end{aligned}$$

where

35 $$\begin{aligned} \mu _k(x):=M_k(0,x)\ge 0. \end{aligned}$$

#### Proposition 4

Let ${\mathcal {L}}$ be given by (32) satisfying (H2) and (H3). Then for any ${\xi }\in {\mathbb {R}}^{N}_+$ there exists a unique solution $w(x)\in C([0,b),{\mathbb {R}}^{N}_+)$ to the initial value problem

36 $$\begin{aligned} \left\{ \begin{array}{rcl} {\mathcal {L}}w(x)&{}=&{}0 \qquad x\in [0,b)\\ w(0) &{}=&{} \xi . \end{array} \right. \end{aligned}$$

The solution is nonnegative and bounded,

37 $$\begin{aligned} 0\le w_k(x)\le \Vert w(0)\Vert _\infty e^{N\Vert {\mathbf {D}}\Vert b}, \end{aligned}$$

and furthermore

38 $$\begin{aligned} \Vert w(x)\Vert _{\infty } \le \Vert w(0)\Vert _{\infty } e^{N\Vert {\mathbf {D}}\Vert b-\int _0^x\mu (s)ds}, \end{aligned}$$

where $$\mu (x)=\min _{k}\mu _k(x).$$

#### Proof

Using the notation of (16), the Metzler property on *D* implies that ${\mathbf {F}}$ satisfies the Kamke–Müller condition in [0, *b*). Furthermore, since $M_k\ge 0$ one also has

$$\begin{aligned} F_k(w,x)\le \Vert w\Vert _{\infty }\sum _{j=1}^N|D_{kj}(x)|\le N\Vert {\mathbf {D}}\Vert \Vert w\Vert _{\infty } \end{aligned}$$

which implies (21) with $C({\mathbf {F}})=N\Vert {\mathbf {D}}\Vert$. Thus, the assumptions of Proposition 2 are fulfilled. This yields the existence of the initial problem (36) and (37). Furthermore, if $H(x)=\Vert w(x)\Vert _{\infty }$ then by (23) at any point $x\in [0,b)$ of differentiability of *H*

$$\begin{aligned} H'(x)&\le \max _{k}F_k(H(x){\mathbf {1}},x)\\&\le N\Vert {\mathbf {D}}\Vert H(x)-\min _{k}M_k(H(x),x)\\&\le (N\Vert {\mathbf {D}}\Vert -\mu (x))H(x) \end{aligned}$$

which readily yields (38). $\square$

#### Proposition 5

(The Universal Majorant) Let ${\mathcal {L}}$ be given by (32) satisfying (H2) and (H3) . Then any solution ${\mathcal {L}}w(x)=0$ satisfies

$$\begin{aligned} w(x) \le \omega _1 x^{-1/\gamma }{\mathbf {1}}_N\quad x\in (0,b), \,\, 1\le k\le N, \end{aligned}$$

where

39 $$\begin{aligned} \omega _1= \left( \frac{1+N\Vert {\mathbf {D}}\Vert b}{\gamma \mu _\infty }\right) ^{1/\gamma }. \end{aligned}$$

#### Proof

Let us consider $h(x)=\omega _1 x^{-1/\gamma }{\mathbf {1}}_N$, where $\omega _1$ is defined by (39). Then using (33) and $M_k(0,x)\ge 0$ we have for any $k=1,\ldots ,N$ and $x\in [0,b)$

$$\begin{aligned} M_k(h_k(x),x)=M_k(\omega _1 x^{-1/\gamma },x)\ge \omega _1^{\gamma }\mu _\infty x^{-1}, \end{aligned}$$

hence

$$\begin{aligned} ({\mathcal {L}}h(x))_{k}&\ge -\frac{\omega _1}{\gamma }x^{-1-1/\gamma } +\mu _\infty \omega _1^{1+\gamma } x^{-1-1/\gamma }-N\Vert {\mathbf {D}}\Vert \omega _1 x^{-1/\gamma }\\&\ge \frac{\omega _1}{\gamma }x^{-1-1/\gamma }(\gamma \mu _\infty \omega _1^{\gamma }-1-N\Vert {\mathbf {D}}\Vert x)\\&\ge \frac{\omega _1}{\gamma }x^{-1-1/\gamma }(\gamma \mu _\infty \omega _1^{\gamma }-1-N\Vert {\mathbf {D}}\Vert b)\\&\ge 0, \end{aligned}$$

i.e. *h*(*x*) is an upper solution. Now, if *w*(*x*) be an arbitrary solution of ${\mathcal {L}}w=0$ then by (22), *w*(*x*) is bounded on [0, *b*): $|w_k(x)|\le \Vert w(0)\Vert _\infty e^{C({\mathbf {F}})b}$ for any $k=1,\ldots ,N$ and $x\in [0,b)$. Since $M_k\ge 0$ one has $C({\mathbf {F}})\le N\Vert {\mathbf {D}}\Vert$. Let $c=\Vert w(0)\Vert _\infty e^{N\Vert {\mathbf {D}}\Vert b}$ and $x_0:=\min \{(\omega _1/ c)^{\gamma },b\}$. Then $h(x)\ge w(x)$ on the whole interval $(0,x_0)$. This proves the claim if $x_0\ge b$. If $x_0<b$ then since *h*(*x*) is an upper solution of (36) and $h(x_0)=c\ge w(x_0)$. Therefore Proposition 1 yields $h(x)\ge w(x)$ for any $x\in (x_0,b)$, which finishes the proof. $\square$

### The main represenation

#### Lemma 4

Let ${\mathscr {B}}$ be defined by (2) and let

$$\begin{aligned} {\mathscr {B}}^-=\{(a,t)\in {\mathscr {B}}:a>t\}, \quad {\mathscr {B}}^+=\{(a,t)\in {\mathscr {B}}:a<t\}. \end{aligned}$$

Then each of ${\mathscr {B}}^-$ and ${\mathscr {B}}^+$ is a connected open set.

#### Proof

It suffices to prove that for any $y\ge 0$, the set $\{s\ge 0: (s,y+s)\in {{\bar{{\mathscr {B}}}}}\}$ is connected. To this end let us suppose that $(0,y)\in {{\bar{{\mathscr {B}}}}}$ and let *S* be the closed component of $\{s\ge 0: (s,y+s)\in {{\bar{{\mathscr {B}}}}}\}$ containing (0, *y*). Let $(s_1,y+s_1)$ be the right endpoint of *S*. Then $(s_1,y+s_1)\in \partial {\mathscr {B}}$. We claim that $(s,y+s)\in {\mathbb {R}}^{2}{\setminus } {{\bar{{\mathscr {B}}}}}$ for $s>s_1$. Indeed, arguing by contradiction, one concludes that there exists $s_2>s_1$ such that $(s_2,y+s_2)\in \partial {\mathscr {B}}$. This yields $B(y+s_i)=s_i$, $i=1,2$, thus by (5)

$$\begin{aligned} 1=\frac{B(y+ks_2)-B(y+ks_1)}{s_2-s_1}<1. \end{aligned}$$

The contradiction yields our claim and, thus, the desired connectedness. $\square$

**Fig. 1:**
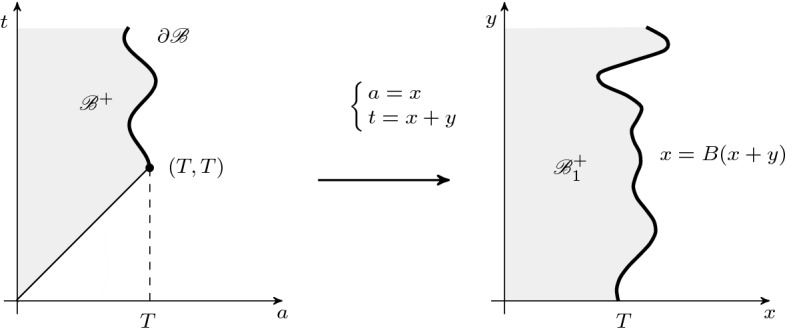
The domains ${\mathscr {B}}^+$ and ${\mathscr {B}}^+_1$

**Fig. 2:**
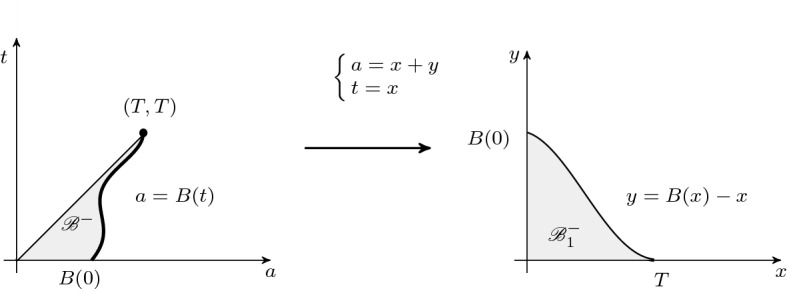
The domains ${\mathscr {B}}^-$ and ${\mathscr {B}}^-_1$

Let us define

$$\begin{aligned} \begin{aligned} {\mathscr {B}}_1^+&=\{(x,y): x<B(x+y),\,y>0\} \\ {\mathscr {B}}_1^-&=\{(x,y): 0<x<T,\,0<y<B(x)-x\}, \end{aligned} \end{aligned}$$

as it is shown on Figs. 1 and  2, respectively.

Next, let $\varPhi (x;\rho ,y)$ denote respectively $\varPsi (x;{\mathbf {f}},y)$ the solutions *h*(*x*) of the initial value problems

40 $$\begin{aligned} \left\{ \begin{aligned} \frac{d }{dx}{h}(x)&=-{\mathbf {M}}({h}(x),x,x+y){h}(x)+{\mathbf {D}}(x,x+y){h}(x), \\ h(0)&= \rho (y),\quad (x,y)\in {\mathscr {B}}_1^+, \end{aligned} \right. \end{aligned}$$

respectively

41 $$\begin{aligned} \left\{ \begin{aligned} \frac{d}{dx}{h}(x)&=-{\mathbf {M}}({h}(x),x+y,x){h}(x) +{\mathbf {D}}(x+y,x){h}(x),\\ h(0)&={\mathbf {f}}(y), \quad (x,y)\in {\mathscr {B}}_1^-. \end{aligned} \right. \end{aligned}$$

#### Lemma 5

Let $\rho \in C({\mathbb {R}}^{}_+,{\mathbb {R}}^{N}_+)\cap L^\infty ({\mathbb {R}}^{}_+,{\mathbb {R}}^{N}_+)$ and let ${\mathbf {f}}\in C({\mathbb {R}}^{}_+,{\mathbb {R}}^{N}_+)$ satisfy (H5). Then $\varPhi (x;\rho ,y)$ (resp. $\varPsi (x;{\mathbf {f}},y)$) is a nonnegative function non-decreasing in $\rho$ (resp. *f*). Furthermore,

42 $$\begin{aligned} \varPhi (x;\rho ,y)&\le e^{N\Vert {\mathbf {D}}\Vert b}\Vert \rho \Vert _\infty \end{aligned}$$

43 $$\begin{aligned} \varPhi (x;\rho ,y)&\le \omega _1 x^{-1/\gamma }{\mathbf {1}}_N , \quad x\ge 0. \end{aligned}$$

where $\omega _1$ is defined by (39), and

44 $$\begin{aligned}&|\varPhi _k(x; \rho ,y)-\varPhi _k(x; \rho ^*,y)|\le e^{N\Vert {\mathbf {D}}\Vert b}\Vert \rho (y)- \rho ^*(y)\Vert _{\infty }, \end{aligned}$$

45 $$\begin{aligned}&\varPsi (x;{\mathbf {f}},y)=0 \quad \forall x\ge 0, y\ge B(0). \end{aligned}$$

#### Proof

It follows from Proposition 4 that (40) and (41) have a unique nonnegative solution. Next, given two arbitrary $\rho$ and $\rho ^*$, let *h*(*x*) and $h^*(x)$ be the corresponding solutions of (40). If $\rho \ge \rho ^*$ then Proposition 1 imply $h(x)\ge h^*(x)$ for $x\ge 0$ and the monotonicity $\varPhi (x;\rho ,y)\ge \varPhi (x; \rho ^*,y)$ follows. Similarly one shows the monotonicity of $\varPsi$. Furthermore, if $\rho (t)$ and $\rho ^*(t)$ are two arbitrary nonnegative vector-functions, then Corollary 3 and Proposition 4 yield

$$\begin{aligned} |\varPhi _k(x; \rho ,y)-\varPhi _k(x; \rho ^*,y)|&=|h(x)-h^*(x)| \le e^{N\Vert {\mathbf {D}}\Vert b}\Vert \rho (y)- \rho ^*(y)\Vert _{\infty }. \end{aligned}$$

Proposition 5 implies (43). Finally, by (H5) $f(x)=0$ for all $x>B(0)$. Then by the uniqueness of solution of (41), $\varPsi (x;{\mathbf {f}},y)=0$ for all $y\ge B(0)$ and $x\ge 0$. $\square$

#### Proposition 6

Let ${\mathbf {n}}(a,t)\in C^1({\overline{{\mathscr {B}}}})$ be a solution to the problem (1)–(4) and let $\rho (t)={\mathbf {n}}(0,t)$. Then

46 $$\begin{aligned} {\mathbf {n}}(a,t)=\left\{ \begin{array}{ll} \varPhi (a; \rho ,t-a), &{}\quad t>a, \\ \varPsi (a; {\mathbf {f}},a-t), &{}\quad a\ge t, \end{array}\right. \end{aligned}$$

and

47 $$\begin{aligned} \rho (t) =\int _0^t{\mathbf {m}}(a,t)\varPhi (a; \rho ,t-a)\,da + \int _t^{\infty }{\mathbf {m}}(a,t)\varPsi (a;{\mathbf {f}},a-t)\,da. \end{aligned}$$

#### Proof

First let $$(a,t)\in {\mathscr {B}}$$ and $$t>a$$. Then in the new variables $$(a,t)=(x,x+y)$$ one has $$(x,y)\in {\mathscr {B}}^+_1$$ and the initial value problem (1)–(4) becomes (40) for $$h(x)={\mathbf {n}}(x,x+y)$$. This yields $${\mathbf {n}}(x,x+y)=\varPhi (x;\rho ,y)$$ for each $$y>0$$, thus, returning to the old variables yields $${\mathbf {n}}(a,t)=\varPhi (a; \rho ,t-a)$$ for any $$ t>a>0$$. This proves the first part of representation (46). The second part is similarly obtained by the change of variables $$(a,t)=(x+y,x)$$. Furthermore, the continuity of $${\mathbf {n}}(a,t)$$ follows from (46) and the standard facts on continuity of solutions on parameters. Finally, plugging (46) in (3) yields (47). $$\square $$

### The integral equation

It is straightforward to see that if ${\mathbf {M}}$, ${\mathbf {D}}$, ${\mathbf {m}}$ and ${\mathbf {f}}$ are sufficiently smooth functions, then the function ${\mathbf {n}}(a,t)$ in (46) is a classical solution of the boundary value problem (1)–(4) in ${\mathscr {B}}$. On the other hand, in application it is natural to assume that these functions are merely continuous (or even measurable). In that case, one can interpret the representation (46) with $\rho$ satisfying (47) as a *weak solution* of (1)–(4). Furthermore, since a solution $\rho (t)$ of the integral Eq. (47) completely determines the population dynamics ${\mathbf {n}}(a,t)$, it is natural to characterize all nonnegative solutions of (47) (with a given function *f*). To this end, we observe that (47) can be thought of as an (nonlinear) operator equation on $\rho$:

48 $$\begin{aligned} \rho ={\mathscr {L}}_{\mathbf {f}}\rho :={\mathcal {K}} \rho + {\mathcal {F}} {\mathbf {f}}, \end{aligned}$$

where the operators ${\mathcal {K}}$ and ${\mathcal {F}}$ are defined resp. by

49 $$\begin{aligned} {\mathcal {K}} \rho (t)&=\int _0^t{\mathbf {m}}(a,t)\varPhi (a; \rho ,t-a)\,da \end{aligned}$$

50 $$\begin{aligned} {\mathcal {F}} {\mathbf {f}}(t)&=\int _t^{\infty }{\mathbf {m}}(a,t)\varPsi (a;{\mathbf {f}},a-t)\,da. \end{aligned}$$

In this section we treat some general properties of ${\mathscr {L}}_{\mathbf {f}}$.

We fix some notation which will be used throughout the remaining part of the paper. Let $\omega _1$ be defined by (39) and let

51 $$\begin{aligned} \omega _2 = \omega _1\Vert {\mathbf {m}}\Vert _\infty \int _{a_m}^{A_m}\frac{da}{a^{1/\gamma }}, \end{aligned}$$

where $A_m, a_m$ are the constants from (H4) and

$$\begin{aligned} {\mathbf {m}}={\mathbf {m}}(a,t)=(m_1(a,t),\ldots ,m_N(a,t)) \end{aligned}$$

is the birth rate. Let us also consider the following subsets of ${\mathbb {R}}^{N}_+$:

52 $$\begin{aligned} \begin{aligned} Q^-&:=\{x\in {\mathbb {R}}^{N}:\,\,\, 0\le x\le \omega _2{\mathbf {1}}_N\},\\ Q^+&:=\{x\in {\mathbb {R}}^{N}:\,\,\, x\ge \omega _2{\mathbf {1}}_N\}. \end{aligned} \end{aligned}$$

#### Lemma 6

Let (H4) be satisfied. Then the operators ${\mathcal {F}}$ and ${\mathcal {K}}$ are positive on the cone of nonnegative continuous vector-functions $C({\mathbb {R}}^{}_+,{\mathbb {R}}^{N}_+)$ and have bounded ranges:

53 $$\begin{aligned} {\mathcal {K}}&:C({\mathbb {R}}^{}_+,{\mathbb {R}}^{N}_+)\rightarrow C({\mathbb {R}}^{}_+, Q^-), \end{aligned}$$

54 $$\begin{aligned} {\mathcal {F}}&:C({\mathbb {R}}^{}_+,{\mathbb {R}}^{N}_+)\rightarrow \{h \in C({\mathbb {R}}^{}_+, Q^-): {{\,\mathrm{\mathrm {supp}}\,}}h\subset [0,A_m]\times {\mathbb {R}}^{}\}, \end{aligned}$$

Furthermore, ${\mathcal {K}}$ is non-decreasing and Lipschitz continuous on $C({\mathbb {R}}^{}_+,{\mathbb {R}}^{N}_+)$.

#### Proof

It readily follows from the nonnegativity of *m* and Lemma 5 that ${\mathcal {K}}$ and ${\mathcal {F}}$ preserve the cone of nonnegative functions $C({\mathbb {R}}^{}_+,{\mathbb {R}}^{N}_+)$ and non-decreasing there. Furthermore, using (H4) we have from (43)

$$\begin{aligned} ({\mathcal {K}} \rho )_k(t) \le \int _{a_m}^{A_m}\frac{\omega _1 m_k(a,t)}{a^{1/\gamma }}\,da \le \omega _1\Vert {\mathbf {m}}\Vert _\infty \int _{a_m}^{A_m}\frac{1}{a^{1/\gamma }}\,da =\omega _2 . \end{aligned}$$

This yields (53) and thus the boundedness of the range of ${\mathcal {K}}$. The corresponding property for ${\mathcal {F}}$ is established similarly. Next, by (H4) ${\mathbf {m}}(a,t)\equiv 0$ for $a\ge A_m$, hence for any $t\ge A_m$

$$\begin{aligned} {\mathcal {F}} {\mathbf {f}}(t)=\int _t^{\infty }{\mathbf {m}}(a,t)\varPsi (a;{\mathbf {f}},a-t)\,da=0 \end{aligned}$$

which implies (54). Finally, if $\rho$ and $\rho ^*$ are bounded functions then by (44),

$$\begin{aligned} |({\mathcal {K}} \rho -{\mathcal {K}} \rho ^*)_k(t)|&\le \Vert {\mathbf {m}}\Vert _{\infty }\int _{a_m}^{A_m}|\varPhi _k(a; \rho , t-a)-\varPhi _k(a; \rho ^*, t-a)|\,da \\&\le (A_m-a_m)\Vert {\mathbf {m}}\Vert _{\infty }e^{N\Vert {\mathbf {D}}\Vert b} \Vert \rho - \rho ^*\Vert _{\infty }, \end{aligned}$$

which yields that ${\mathcal {K}}$ is a Lipschitz continuous operator. $\square$

#### Proposition 7

Given an arbitrary ${\mathbf {f}}\in C({\mathbb {R}}^{}_+,{\mathbb {R}}^{N}_+)$, there exists a unique solution $\rho \in C({\mathbb {R}}^{}_+,{\mathbb {R}}^{N}_+)\cap L^\infty ({\mathbb {R}}^{}_+,{\mathbb {R}}^{N}_+)$ of (48).

#### Proof

Let us consider the sequence $\{\rho ^{(i)}\}_{0\le i\le \infty }$ defined recursively by

55 $$\begin{aligned} \rho ^{(i+1)}= {\mathcal {K}}\rho ^{(i)}+{\mathcal {F}} {\mathbf {f}}, \quad \rho ^{(0)}=0. \end{aligned}$$

Since ${\mathcal {F}} {\mathbf {f}}\ge 0$, we have

$$\begin{aligned} \rho ^{(0)}&=0 \le {\mathcal {F}} {\mathbf {f}}= \rho ^{(1)}, \\ \rho ^{(1)}&= {\mathcal {F}} {\mathbf {f}} \le {\mathcal {K}} \rho ^{(1)} +{\mathcal {F}} {\mathbf {f}}= \rho ^{(2)}. \end{aligned}$$

This shows that $\rho ^{(i+1)}-\rho ^{(i)}\ge 0$ for $i=0,1$. Then combining

$$\begin{aligned} \rho ^{(i+1)}-\rho ^{(i)}&= {\mathcal {K}} \rho ^{(i)} -{\mathcal {K}}\rho ^{(i-1)} , \quad i\ge 0, \end{aligned}$$

with the monotonicity of ${\mathcal {K}}$ implies by induction that $\rho ^{(i+1)}-\rho ^{(i)} \ge 0$ for any $i\ge 0.$ In other words, $\{\rho ^{(i)}\}_{0\le i\le \infty }$ is a pointwise non-decreasing sequence. On the other hand, by (53) and (54) this sequence is uniformly bounded:

$$\begin{aligned} \rho ^{(i+1)}= {\mathcal {K}}\rho ^{(i)}+{\mathcal {F}} {\mathbf {f}}\le 2\omega _2\cdot \mathbf{1 }_N. \end{aligned}$$

This implies the the existence of the limit

56 $$\begin{aligned} \rho :=\lim _{i\rightarrow \infty }\rho ^{(i)}\le 2\omega _2\cdot \mathbf{1 }_N. \end{aligned}$$

Using (44) and (42) we obtain for any $i\ge 1$

$$\begin{aligned} |\rho ^{(i+1)}_k(t) - \rho ^{(i)}_k(t)|&\le \Vert {\mathbf {m}}\Vert _{\infty } \int \limits _0^t |\varPhi _k(a; \rho ^{(i)},t-a)-\varPhi _k(a; \rho ^{(i-1)},t-a)|\,da\\&\le C \int \limits _0^t |\rho ^{(i)}(t-a)-\rho ^{(i-1)}(t-a)|\,da\\&= C \int \limits _0^t |\rho ^{(i)}(a)-\rho ^{(i-1)}(a)|\,da, \end{aligned}$$

where $C=e^{Nb\Vert {\mathbf {D}}\Vert } \Vert {\mathbf {m}}\Vert _{\infty }$. On iterating the latter inequality we obtain using $\rho ^{(1)}\le \rho$ and (56)

$$\begin{aligned} |\rho ^{(i+1)}_k(t)- \rho ^{(i)}_k(t)| \le C^i \int \limits _0^t\int \limits _0^{a_1}\ldots \int \limits _0^{a_{i-1}} \rho ^{(1)}(a)da\,da_1\ldots \,da_{i-1} \le 2\omega _2\frac{C^it^i}{i!} \end{aligned}$$

therefore

57 $$\begin{aligned} |\rho ^{(i+j)}_k(t)-\rho ^{(i)}_k(t)| \le 2\omega _2\sum _{s=0}^{j-1}\frac{(Ct)^{i+s}}{(i+s)!} \le 2\omega _2 e^{Ct}\frac{C^it^i}{i!}. \end{aligned}$$

Therefore for any fixed $T>0$ and $0<t<T$, the latter expression converges to 0 as $i\rightarrow \infty$ uniformly in $j\ge 1$. This establishes that $\rho ^{(i)}\rightarrow \rho$ in $L^{\infty }((0,T), {\mathbb {R}}^{N}_+)$ for each $T>0$. In particular, by (55) this implies that $\rho$ satisfies (48).

In order to establish the uniqueness we assume that $\rho$ and ${\tilde{\rho }}$ are two solutions to (48). The tautological iterations $\rho ^{(i)}:=\rho$ and ${\tilde{\rho }}^{(i)}:={\tilde{\rho }}$, $i=0,1,2,\ldots$ obviously satisfy (55) which by virtue of (57) yields

$$\begin{aligned} |\rho _k(t)-{\tilde{\rho }}_k(t)| \le 2\omega _2\frac{C^it^i}{i!}\rightarrow 0 \quad \text{ as }\quad i\rightarrow \infty , \end{aligned}$$

thus $\rho (t)\equiv {\tilde{\rho }}(t)$. Finally, by Lemma 5$\varPhi _k$ and $\varPsi _k$ are continuous, which yields the continuity of operators ${\mathcal {K}}$ and ${\mathcal {F}}$, and, thus, all iterations given by (55) are continuous and so is the limit $\rho$. This completes the proof. $\square$

### The convolution property of ${\mathcal {K}}$

#### Lemma 7

Let $\rho \in C({\mathbb {R}}^{}_+,{\mathbb {R}}^{N}_+)$ and $\rho (t)>0$ for $t\in [s_1,s_2]\subset {\mathbb {R}}^{}_+$. Let for some *k* there exists $\beta _k<\sup {{\,\mathrm{\mathrm {supp}}\,}}m_k$ such that the patch *k* is accessible at $\beta _k$. Then there exist $a_k,b_k$ such that $[a_k,b_k]\Subset {{\,\mathrm{\mathrm {supp}}\,}}m_k$, $\beta _k\le a_k$, and $({\mathcal {K}}\rho (t))_k> 0$ for all $t\in [s_1+a_k,s_2+b_k]$.

#### Proof

There are $\delta >0$ and points $a_k',b_k'$, $a_k'<a_k<b_k<b_k'$, such that (i) $m_k(a)\ge \delta$ for $a\in [a_k',b_k']$, and (ii) the patch *k* is accessible at $\beta _k\le a_k$. By Lemma 3 we have

$$\begin{aligned} \delta _1:=\min _{\begin{array}{c} s_1 \le y\le s_2 \\ a_k' \le a\le b_k' \end{array}} \varPhi _k(a;\rho ,y)>0, \end{aligned}$$

hence if $t\in [s_1+a_k,s_2+b_k]$ then

$$\begin{aligned} ({\mathcal {K}}\rho )_k(t)&= \int _0^tm_k(a)\varPhi _k(a; \rho ,t-a)\,da \ge \delta \int _{a_k'}^{\min \{t,b_k'\}}\varPhi _k(a; \rho ,t-a)\,da\\&\ge \delta \delta ' \int _{\max \{a_k',t-s_2\}}^{\min \{t,b_k',t-s_1\}}\,da =\delta \delta ' \int _{\max \{a_k',t-s_2\}}^{\min \{b_k',t-s_1\}}\,da \end{aligned}$$

We claim that $({\mathcal {K}}\rho (t))_k> 0$ for all $t\in [s_1+a_k,s_2+b_k]$. Indeed, the function $\xi (t)=\min \{b_k',t-s_1\}-\max \{a_k',t-s_2\}$ is obviously concave and

$$\begin{aligned} \xi (s_1+a_k')&=\min \{b_k',a_k'\}-\max \{a_k',a_k'+s_1-s_2\}=0,\\ \xi (s_2+b_k')&=\min \{b_k',b_k'+s_2-s_1\}-\max \{a_k',b_k'\}=0, \end{aligned}$$

hence by the maximum principle $\xi (t)>0$ for any $t\in (s_1+a_k',s_2+b_k')$. This yields the desired conclusion. $\square$

## Constant environment

The model (1)–(4) is more complicated for analysis under the assumption that a population lives in a temporally variable environment because the structure parameters are functions of age and time. In this section we analyze a constant environment, then in Sect. 6 we continue with a periodically changing environment, and finally in Sect. 7 we describe an irregularly changing environment. Throughout this section, we assume the conditions (H1)–(H5) are fulfilled and that the maximal life-time is constant: $B(t)\equiv b$. This condition is natural and is commonly used for both finite and infinite values of *b*, see Chipot (1983), Cushing (1984) and Gurtin and MacCamy (1974).

### The characteristic equation

Under assumptions that the vital rates, carrying capacity and dispersion coefficients are *time-independent* functions, the system (1)–(4) becomes

58 $$\begin{aligned} \left\{ \begin{aligned}&\frac{\partial \mathbf{n }(a,t)}{\partial t}+\frac{\partial \mathbf{n }(a,t)}{\partial a} = -\mathbf{M }(\mathbf{n }(a,t),a) \mathbf{n }(a,t) +\mathbf{D }(a)\mathbf{n }(a,t), \\&\mathbf{n }(0,t)=\int _0^{\infty }\mathbf{m }(a)\mathbf{n }(a,t)\,da, \\&\mathbf{n }(a,0) =\mathbf{f }(a). \end{aligned} \right. \end{aligned}$$

According to Proposition 6, there exists a unique solution ${\mathbf {n}}(a,t)$ of the problem (58) given by

59 $$\begin{aligned} {\mathbf {n}}(a,t)=\left\{ \begin{array}{ll} \varPhi (a; \rho ,t-a), &{}\quad a<t, \\ \varPsi (a;{\mathbf {f}},a-t), &{}\quad a\ge t, \end{array}\right. \end{aligned}$$

where the *newborns function*

$$\begin{aligned} \rho (t)\equiv (\rho _1(t),\ldots ,\rho _N(t))^t={\mathbf {n}}(0,t)=\int _0^{\infty }{\mathbf {m}}(a){\mathbf {n}}(a,t)\,da, \end{aligned}$$

satisfies the following identity:

60 $$\begin{aligned} {\rho }(t)=\int _0^t{\mathbf {m}}(a){\varPhi }(a;\rho ,t-a)\,da +\int _t^{\infty }{\mathbf {m}}(a){\varPsi }(a;{\mathbf {f}},a-t)\,da. \end{aligned}$$

Using the notation of (49) and (50), we have

#### Proposition 8

Let ${\mathbf {n}}(a,t)$ be the solution of the problem (58). Then the newborns function $\rho (t)$ satisfies the integral equation

61 $$\begin{aligned} \rho ={\mathscr {L}}_{\mathbf {f}}\rho := {\mathcal {K}}\rho +{\mathcal {F}}{\mathbf {f}}. \end{aligned}$$

It is natural to study stationary (i.e. time independent) solutions of (61). Indeed, since *m*(*a*) has a compact support, it follows from (60) that ${\mathcal {F}}{\mathbf {f}}$ vanishes for large enough *t*. This yields that any solution of (61) satisfies

62 $$\begin{aligned} \rho (t)=({\mathcal {K}}\rho )(t)\quad \text {for all}\quad t\ge A_m. \end{aligned}$$

In particular, it is easy to see that if $\rho$ has a limit $\rho _\infty =\lim _{t\rightarrow \infty }\rho (t)$ then $\rho _\infty$ itself is a stationary solution of (62). In the next section we study the stationary solutions in more detail.

To make these observations precise, we introduce the following operator:

63 $$\begin{aligned} \bar{{\mathcal {K}}}\rho :=\int _0^{\infty }{\mathbf {m}}(a)\varphi (a;\rho )\,da \equiv \int _{a_m}^{A_m}{\mathbf {m}}(a)\varphi (a;\rho )\,da , \quad \rho \in {\mathbb {R}}^{N}_+, \end{aligned}$$

where $\varphi (a;\rho )=(\varphi _1(a;\rho ), \ldots , \varphi _N(a;\rho ))^t$ is the unique solution of the initial problem

64 $$\begin{aligned} \left\{ \begin{aligned} \frac{d \varphi (a;\rho )}{da}&=-{\mathbf {M}}(\varphi (a;\rho ),a)\varphi (a;\rho ) +{\mathbf {D}}(a)\varphi (a;\rho ),\\ \varphi (0;\rho )&=\rho . \end{aligned} \right. \end{aligned}$$

In particular, this yields in the notation of (40) for any $\rho \in {\mathbb {R}}^{N}_+$ that

65 $$\begin{aligned} \varphi (a;\rho ) \equiv \varPhi (a;\rho ,y)\quad \text {for any}\quad y\in {\mathbb {R}}^{}. \end{aligned}$$

#### Corollary 4

The operator $\bar{{\mathcal {K}}}$ is nondecreasing and

66 $$\begin{aligned} \bar{{\mathcal {K}}}:{\mathbb {R}}^{N}_+\rightarrow Q^-, \end{aligned}$$

where $Q^-$ is defined by (52).

#### Proof

The nondecreasing property is by Proposition 1 and (66) follows from (53). $\square$

#### Definition 2

The equation

67 $$\begin{aligned} \bar{{\mathcal {K}}}\rho =\rho . \end{aligned}$$

is said to be the *characteristic equation* for the problem (61). A nonnegative solution $\rho$ of (67) is called a *stationary solution* of (61).

The set of stationary solutions is nonempty because $\rho =0$ is a (trivial) stationary solution. In Sect. 5.2 we characterize all nontrivial stationary solutions.

As it was noticed before, the characteristic equation describes the possible scenario of the limit at infinity of solutions to (61). The next lemma makes this observation more precise. First let us note that the limit

$$\begin{aligned} \rho _\infty :=\rho (M)\equiv \lim _{t\rightarrow \infty }\rho (t). \end{aligned}$$

is well-defined for any $\rho \in S_M$, where

$$\begin{aligned} S_M:=\{\rho :{\mathbb {R}}^{}_+\rightarrow {\mathbb {R}}^{N}_+ \text { such that } \rho (t) \text { is constant for }t\ge M\}. \end{aligned}$$

#### Lemma 8

For any $$f\in C({\mathbb {R}}^{}_+,{\mathbb {R}}^{N}_+)$$,

$$\begin{aligned} {\mathscr {L}}_{\mathbf {f}}:S_M\rightarrow S_{M+A_m} \end{aligned}$$

and for any $\rho \in S_M$

68 $$\begin{aligned} ({\mathscr {L}}_{\mathbf {f}}\rho )_\infty =\bar{{\mathcal {K}}}\rho _\infty . \end{aligned}$$

#### Proof

It follows from (54) and (H4) that for any $t\ge M+A_m$ there holds

$$\begin{aligned} {\mathscr {L}}_{\mathbf {f}}\rho (t)={\mathcal {K}}\rho (t)&= \int _0^t{\mathbf {m}}(a)\varPhi (a; \rho ,t-a)\,da=\int _0^{A_m}{\mathbf {m}}(a)\varPhi (a; \rho ,t-a)\,da. \end{aligned}$$

Next, by virtue our choice of *t* we have for any $0\le a\le A_m$ that $t-a\ge t-A_m\ge M$, therefore $\varPhi (a; \rho ,t-a)= \varPhi (a;\rho _\infty ,M)=\varphi (a;\rho _\infty )$. Therefore for all $t\ge M+A_m$

$$\begin{aligned} {\mathscr {L}}_{\mathbf {f}}\rho (t)=\int _0^{A_m}{\mathbf {m}}(a)\varPhi (a;\rho _\infty ,M) \,da\equiv \int _0^{\infty }{\mathbf {m}}(a)\varphi (a; \rho _\infty )\,da=\bar{{\mathcal {K}}}\rho _\infty \end{aligned}$$

which yields the desired conclusions. $\square$

### The maximal solution of the characteristic equation

A vector $\rho \in {\mathbb {R}}^{N}_+$ is called an *upper* (resp. *lower*) solution to Eq. (67) if $\rho \ge \bar{{\mathcal {K}}}\rho$ (resp. $\rho \le \bar{{\mathcal {K}}}\rho$).

#### Lemma 9

The set of lower solutions of (67) is bounded:

$$\begin{aligned} \{\rho :\bar{{\mathcal {K}}}\rho \le \rho \}\subset Q^-. \end{aligned}$$

Furthermore, any $\rho \in Q^+$ is an upper solution of (67).

#### Proof

Indeed, if $\rho \le \bar{{\mathcal {K}}}\rho$ then applying (67), (43) and (51) one obtains

$$\begin{aligned} \rho \le \int _0^{\infty }{\mathbf {m}}(a)\varphi (a;\rho )\,da \le \int _{a_m}^{A_m}{\mathbf {m}}(a){\mathbf {1}}_N\frac{\omega _1}{a^{1/\gamma }}\,da \le \omega _2{\mathbf {1}}_N \end{aligned}$$

which yields $\rho \in Q^-$, and therefore the first claimed inclusion. Next, arguing similarly we have for any $\rho \in Q^+$ that

$$\begin{aligned} \rho \ge \omega _2\cdot \mathbf{1 }_{N}\ge \int _0^{\infty }{\mathbf {m}}(a)\varphi (a;\rho )\,da=\bar{{\mathcal {K}}}\rho \end{aligned}$$

which proves that $\rho$ is an upper solution of (67). $\square$

#### Proposition 9

For any $\rho ^+\in Q^+$ the limit

69 $$\begin{aligned} \theta :=\lim _{i\rightarrow \infty }\bar{{\mathcal {K}}}^i\rho ^+ \end{aligned}$$

exists and $\theta$ is a solution of the characteristic equation. Furthermore,

(i) $\theta$ does not depend on a particular choice of $\rho ^+\in Q^+$;
(ii) if $\rho$ is an arbitrary lower solution of (67) then $\rho \le \theta$.

#### Proof

By Lemma 9, $\bar{{\mathcal {K}}}\rho ^+\le \rho ^+$. Thus, by the monotonicity of $\bar{{\mathcal {K}}}$ we have for all $i\ge 0$ that

$$\begin{aligned} \bar{{\mathcal {K}}}^{i+1}\rho \equiv \bar{{\mathcal {K}}}^i\bar{{\mathcal {K}}}\rho ^+\le \bar{{\mathcal {K}}}^i \rho ^+, \end{aligned}$$

thus $\{\bar{{\mathcal {K}}}^i \rho ^+\}$ is a non-increasing sequence bounded from below: $\bar{{\mathcal {K}}}^i \rho ^+\ge 0$. This implies the existence of the limit in (69). Let us for a moment denote the limit by $\theta (\rho ^+)$. It follows trivially that $\bar{{\mathcal {K}}}\theta (\rho ^+)=\theta (\rho ^+)$. This proves that $\theta (\rho ^+)$ is a solution of the characteristic equation. Next, let $\rho$ be an arbitrary lower solution of (67). Then by Lemma 9

$$\begin{aligned} \rho \le \bar{{\mathcal {K}}}\rho \le \omega _2 \mathbf{1 }_{N}\le \rho ^+. \end{aligned}$$

Iterating the latter inequality yields $\rho \le \bar{{\mathcal {K}}}^i \rho \le \bar{{\mathcal {K}}}^i \rho ^+$, and passing to the limit as $i\rightarrow \infty$ we get $\rho \le \rho ^+(\theta )$. This proves (ii). Now suppose that $\rho ^+_1\in Q^+$. Then $\theta (\rho ^+_1)$ is a solution of the characteristic equation, hence by (ii)

$$\begin{aligned} \theta (\rho ^+_1)\le \theta (\rho ^+), \end{aligned}$$

which, by symmetry, yields the equality in the latter inequality. This establishes the independence of $\theta (\rho ^+)$ on a choice of $\rho ^+$, implying (i). $\square$

#### Definition 3

The unique $\theta$ defined by (69) is called the *maximal solution* of the characteristic equation.

Note that the maximal solution $\theta$ does not depend on the initial population distribution ${\mathbf {f}}(a)$ and it is essentially determined by the maternity function ${\mathbf {m}}(a)$. As we shall see, the maximal solution plays a distinguished role in the asymptotic analysis.

### The basic reproduction number dichotomy

Throughout this section we assume additionally that the condition (H6) is also fulfilled. Let us consider the scaled version of $\bar{{\mathcal {K}}}$ by

70 $$\begin{aligned} {\mathscr {R}}_\lambda x=\frac{1}{\lambda } \bar{{\mathcal {K}}}\lambda x, \quad x\in {\mathbb {R}}^{N}_+, \quad \lambda \in (0,\infty ). \end{aligned}$$

Equivalently, we have component-wise

71 $$\begin{aligned} {\mathscr {R}}_\lambda x:=\int _0^{\infty }{\mathbf {m}}(a){\mathbf {Y}}(a; x,\lambda )\,da, \end{aligned}$$

where

$$\begin{aligned} {\mathbf {Y}}(a; x , \lambda )=\frac{1}{\lambda }\varphi (a;\lambda x), \quad x\in {\mathbb {R}}^{N}_+. \end{aligned}$$

Thus, the existence of a nontrivial solution to the characteristic Eq. (67) is equivalent to the existence of a pair $(e,\lambda )$ , where a unit vector (a direction) $e\in {\mathbb {R}}^{N}_+$, $\Vert e\Vert =1$ and a scalar $\lambda >0$ are such that

72 $$\begin{aligned} e ={\mathscr {R}}_\lambda e. \end{aligned}$$

The next lemma establishes that for each direction $e\in {\mathbb {R}}^{N}_+$ there is at most one such pair. We denote by ${\mathscr {C}}$, ${\mathscr {C}}^{\mathrm {up}}$ and ${\mathscr {C}}^{\mathrm {low}}$ the classes of solutions, upper and lower solutions of (67), respectively.

#### Lemma 10

The operator ${\mathscr {R}}_\lambda$ is decreasing with respect to $\lambda$:

73 $$\begin{aligned} \lambda _2>\lambda _1\ge 0 \quad \Rightarrow \quad {\mathscr {R}}_{\lambda _1}x\gg {\mathscr {R}}_{\lambda _2}x\quad \forall x\in {\mathbb {R}}^{N}_+. \end{aligned}$$

In particular, given an arbitrary direction $e\in {\mathbb {R}}^{N}_+$, $\Vert e\Vert =1$,

$$\begin{aligned} \mathrm {card}\{\lambda>0:\,\lambda e\in {\mathscr {C}}\}\equiv \mathrm {card}\{\lambda >0:\, e={\mathscr {R}}_{\lambda } e\}\le 1, \end{aligned}$$

where $\mathrm {card}$ is the cardinality of the corresponding set.

#### Proof

Since $\alpha =\lambda _2/\lambda _1>1$ we have from (28)

$$\begin{aligned} \varphi (a;\lambda _2 x)=\varphi (a;\alpha \lambda _1 x)\le \alpha \varphi (a; \lambda _1 x), \end{aligned}$$

i.e. $Y(a;x, \lambda _2)\le Y(a;x, \lambda _1)$. This yields the weaker inequality ${\mathscr {R}}_{\lambda _1}x\ge {\mathscr {R}}_{\lambda _2}x$ for any $x\in {\mathbb {R}}^{N}_+$. Next, by (H6) for an arbitrary $1\le k\le N$, there exists $\beta _k\le \sup {{\,\mathrm{\mathrm {supp}}\,}}m_k$ such that the patch *k* is accessible at $\beta _k$. By (29), $\varphi _k(a;\alpha \lambda _1 x)< \alpha \varphi (a; \lambda _1 x)$ holds for any $a>\beta _k$. Thus, $Y_k(a;x, \lambda _2)< Y_k(a;x, \lambda _1)$ for $a>\beta _k$. Since ${{\,\mathrm{\mathrm {supp}}\,}}m_k(a)\cap (\beta _k,\infty )$ has an nonempty interior, it follows from (71) that $({\mathscr {R}}_{\lambda _1}x)_k> ({\mathscr {R}}_{\lambda _2}x)_k$ for any $x\in {\mathbb {R}}^{N}_+$. By the arbitrariness of *k* one has (73). Next, $e\in {\mathbb {R}}^{N}_+$, $\Vert e\Vert =1$ be such that the set $\{\lambda >0:\, e={\mathscr {R}}_{\lambda } e\}$ is nonempty, say $e={\mathscr {R}}_{\lambda _0} e$ for some $\lambda _0>0$. Then (73) yields

$$\begin{aligned} {\mathscr {R}}_{\lambda _2} e\ll e={\mathscr {R}}_{\lambda _0} e \ll {\mathscr {R}}_{\lambda _1} e \end{aligned}$$

for any $\lambda _1<\lambda _0<\lambda _2$. This proves that $\lambda _0$ is the only solution of $e={\mathscr {R}}_{\lambda } e$. $\square$

In the course of the proof of the lemma we have established the following property.

#### Corollary 5

For any $0<\lambda <1$ and any $x\in {\mathbb {R}}^{N}_+$ there holds $\lambda \varphi (a;x)\le \varphi (a; \lambda x$).

The limit case $\lambda =0$ plays a distinguished role in the further analysis. Notice that $Y_k(a;x,\lambda )$ is non-decreasing in $\lambda >0$ and by (42) $Y_k(a;x,\lambda )\le e^{N\Vert {\mathbf {D}}\Vert b}$, where the constant *b* is from (H1). This implies that the limit

$$\begin{aligned} Y_k(a;x):=\lim _{\lambda \rightarrow +0}Y_k(a;x,\lambda ) \end{aligned}$$

does exist for any fixed $x\in {\mathbb {R}}^{N}_+$, and the standard argument shows that ${\mathbf {Y}}(a;x)$ is the unique solution of the *linear* system

74 $$\begin{aligned} \left\{ \begin{aligned} \frac{d {\mathbf {Y}}(a;x)}{da}&=({\mathbf {D}}(a)-{\mathbf {M}}(0,a)){\mathbf {Y}}(a;x), \\ {\mathbf {Y}}(0;x)&=x. \end{aligned} \right. \end{aligned}$$

Here

$$\begin{aligned} {\mathbf {M}}(0,a)={{\,\mathrm{\mathrm {diag}}\,}}(\mu _1(a),\ldots , \mu _N(a)) \end{aligned}$$

with $\mu _k(x)$ is defined by (35). Since $m_k\ge 0$, the limit

75 $$\begin{aligned} {\mathscr {R}}_{0}x=\lim _{\lambda \rightarrow +0} \int _0^{\infty }{\mathbf {m}}(a){\mathbf {Y}}(a;x,\lambda )\,da= \int _0^{\infty }{\mathbf {m}}(a){\mathbf {Y}}(a;x)\,da. \end{aligned}$$

is well defined for each $x\in {\mathbb {R}}^{N}_+$.

To proceed, we recall some standard concepts of the nonnegative matrix theory. A matrix *A* is called reducible (Minc 1988) if for some permutation matrix *P*

$$\begin{aligned} P A P^t= \left( \begin{array}{ccc} A_{11} &{} 0 \\ A_{21} &{} A_{22} \\ \end{array} \right) , \end{aligned}$$

where $A_{11}, A_{22}$ are square matrices, otherwise *A* is called irreducible. There is the following combinatorial characterization of the irreducibility, see Berman and Plemmons (1979, p. 27), Meyer (2000, p. 671): the condition that a *nonnegative* matrix *A* of order $n\ge 2$ is irreducible is equivalent to any of the following conditions:

- no nonnegative eigenvector of *A* has a zero coordinate;
- *A* has exactly one (up to scalar multiplication) nonnegative eigenvector, and this vector is positive;
- $$\alpha x\ge Ax$$ and $$x>0$$ implies $$x\gg 0$$;
- the associated graph $\varGamma (A)$ is strongly connected.

#### Lemma 11

The map ${\mathscr {R}}_{0}: {\mathbb {R}}^{N}\rightarrow {\mathbb {R}}^{N}$ defined by (75) is linear and strongly positive, i.e. $x>0$ implies ${\mathscr {R}}_{0}x\gg 0$. In particular, ${\mathscr {R}}_{0}$ is an irreducible matrix. Furthermore,

76 $$\begin{aligned} {\mathscr {R}}_{\lambda }x \ll {\mathscr {R}}_{0} x, \quad \forall x\in {\mathbb {R}}^{n}_+, \,\,\lambda >0. \end{aligned}$$

#### Proof

Indeed, the linearity follows immediately by (75) and (74). Since the matrix ${\mathbf {M}}(0,a)$ is diagonal, the associated digraphs of the matrices ${\mathbf {D}}(a)$ and ${\mathbf {D}}(a)-{\mathbf {M}}(0,a)$ are equal. Therefore, using (H6) readily yields that $Y_k(a;x,0)> 0$ for any $a>\beta _k$. Hence, repeating the argument of Lemma 10 we have from (75) and (H4) that $({\mathscr {R}}_{0}x)_k>0$ for any *k*. This proves ${\mathscr {R}}_{0}x\gg 0$. Suppose by contradiction that ${\mathscr {R}}_{0}$ is reducible. Then for some permutation matrix *P*

77 $$\begin{aligned} P {\mathscr {R}}_{0} P^t= \left( \begin{array}{ccc} A_{11} &{} 0 \\ A_{21} &{} A_{22} \\ \end{array} \right) , \end{aligned}$$

where $A_{11}, A_{22}$ are square matrices. Let $x>0$ be a vector in ${\mathbb {R}}^{N}_+$ with all first *m* coordinates zero, where *m* is the order of $A_{11}$. By (77) $P {\mathscr {R}}_{0} P^t x$ has the same property, i.e. the vector ${\mathscr {R}}_{0}P^t x$ has at least *m* zero coordinates which contradicts to the fact that ${\mathscr {R}}_{0}P^t x\gg 0$. This proves the irreducibility. Finally, (76) follows from (73). $\square$

#### Corollary 6

If ${\mathscr {R}}_{0} e\le e$ for any $e\in {\mathbb {R}}^{N}_+$, $\Vert e\Vert =1$, then the characteristic Eq. (67) admits only trivial solutions.

#### Proof

Indeed, if $\rho \ne 0$ is a nontrivial solution of (67) then by (70) $e=\rho /\Vert \rho \Vert$ is a solution of ${\mathscr {R}}_{\lambda }e =e$ for $\lambda =\Vert \rho \Vert$. On the other hand, using the assumption and (76) we obtain

$$\begin{aligned} e={\mathscr {R}}_{\lambda }e \ll {\mathscr {R}}_{0} e\le e, \end{aligned}$$

a contradiction follows. $$\square $$

Let us denote by $R_0$ the spectral radius of the linear map ${\mathscr {R}}_{0}$. Combining the irreducibility of ${\mathscr {R}}_{0}$ with the Perron–Frobenius theorem (Berman and Plemmons 1979, Theorem 1.3.26) implies the following important observation.

#### Corollary 7

The spectral radius $R_0>0$ and it is a simple eigenvalue of ${\mathscr {R}}_{0}$. If *x* is an eigenvector of ${\mathscr {R}}_{0}$ then $x\gg 0$. If $\lambda \ne R_0$ is another eigenvalue of ${\mathscr {R}}_{0}$ then $|\lambda |<R_0$. Furthermore, the Collatz-Wielandt identity holds

$$\begin{aligned} \max _{x>0} \min _{\begin{array}{c} 1\le i\le N\\ x_i\ne 0 \end{array}} \frac{({\mathscr {R}}_{0}x)_i}{x_i}=R_0. \end{aligned}$$

#### Definition 4

The linear map ${\mathscr {R}}_{0}$ is called the *net reproductive map* associated to the problem (58). Its spectral radius $R_0$ is called the *basic reproduction number*.

The latter definition can be motivated as folows. For a single patch model, i.e. $N=1$, the linear system (74) becomes a single equation

$$\begin{aligned} \frac{d}{da}Y_1(a;x,0)=-\mu (a)Y_1(a;x,0), \end{aligned}$$

with an explicit solution $Y_1(a;x,0)=x\exp (-\int _{0}^a \mu (s)ds)$. Thus (75) yields

78 $$\begin{aligned} {\mathscr {R}}_{0}x=R_0x, \end{aligned}$$

where

79 $$\begin{aligned} R_0=\int _0^{\infty }m(a)e^{-\int _0^a\mu (s)ds}\,da. \end{aligned}$$

The quantity $R_0$ is well-established and is known as the (inherent) basic reproduction number in the linear time-independent model on a single patch (Iannelli 1995; Cushing 1998); see also Kozlov et al. (2016b) or Kozlov et al. (2017). Note that in this case,

80 $$\begin{aligned} \varPi (a)=e^{-\int _0^a\mu (s)ds} \end{aligned}$$

is the survival probability, i.e. the probability for an individual to survive to age *v*. Then $R_0$ is the expected number of offsprings per individual per lifetime. Recall that in the one-dimensional case, $R_0$ is related to the intrinsic growth rate of population by the characteristic equation. Namely, when $R_0>1$ population is growing, while for $R_0\le 1$ population is declining. The next result extends this dichotomy onto the general multipatch case.

#### Theorem 1

(The Net Reproductive Rate Dichotomy) If $R_0\le 1$ then $\theta = 0$ and the Eq. (67) has no nontrivial solutions. If $R_0> 1$ then $\theta \gg 0$ and $\theta$ is the only nontrivial solution of the characteristic Eq. (67).

#### Proof

First let us assume that $R_0\le 1$ and suppose by contradiction that $\bar{{\mathcal {K}}}\rho =\rho$ for some $\rho >0$. Let $\lambda =\Vert \rho \Vert$ and $e=\rho /\lambda$, then by (70) and (76),

$$\begin{aligned} {\mathscr {R}}_0e\gg {\mathscr {R}}_\lambda e=\frac{1}{\lambda } \bar{{\mathcal {K}}}\lambda e=\frac{1}{\lambda }\bar{{\mathcal {K}}}\rho =\frac{1}{\lambda }\rho =e. \end{aligned}$$

The latter easily implies that there exists $t>1$ such that ${\mathscr {R}}_0e\ge te$. On iterating the obtained inequality yields ${\mathscr {R}}_0^k e\ge t^ke$, thus

$$\begin{aligned} R_0=\lim _{k\rightarrow \infty }\Vert {\mathscr {R}}_0^k\Vert ^{1/k}\ge t>1, \end{aligned}$$

a contradiction.

Now suppose that $R_0>1$. By Corollary 7, there exists a positive eigenvector $e_0\gg 0$ of ${\mathscr {R}}_{0}$. Since $e_0\gg 0$ there exists $\lambda >0$ such that $\lambda e_0\ge \omega _2{\mathbf {1}}_N$, where $\omega _2$ is defined by (51). By (52), $\rho ^+:=\lambda e_0\in Q^+$, hence Lemma 9 implies that

$$\begin{aligned} \theta =\lim _{i\rightarrow \infty }\bar{{\mathcal {K}}}^i\rho ^+\in {\mathscr {C}}\end{aligned}$$

is a solution to (67). On the other hand, since $R_0>1$ we have

$$\begin{aligned} {\mathscr {R}}_{0}e_0=R_0e_0\gg e_0. \end{aligned}$$

hence, by the continuity argument for some $\lambda >0$ small enough there holds

$$\begin{aligned} {\mathscr {R}}_{\lambda }e_0\gg e_0. \end{aligned}$$

Therefore, setting $\rho ^-:=\lambda e_0$ we obtain

$$\begin{aligned} \bar{{\mathcal {K}}}\rho ^-=\bar{{\mathcal {K}}}\lambda e_0=\lambda {\mathscr {R}}_{\lambda }e_0\gg \lambda e_0=\rho ^-, \end{aligned}$$

i.e. $\rho ^-$ is an lower solution of (67). In other words, $\rho ^-\in {\mathscr {C}}^{\mathrm {low}}$, thus (ii) of Proposition 9 yields

$$\begin{aligned} \theta \ge \rho ^-\gg 0, \end{aligned}$$

thus $\theta$ is a nontrivial solution.

In order to establish the uniqueness of a nontrivial solution (i.e. that $\mathrm {card} ({\mathscr {C}})=1$), we will follow the idea of Krasnoselskii and Zabreiko from Krasnosel’skiĭ and Zabreĭko (1984), Ch. 6. To this end, let us suppose that $\theta _1,\theta _2$ be two nontrivial solutions to (67). Then $\theta _1,\theta _2\gg 0$. If $\theta _1\ne \theta _2$ then at least one of inequalities $\theta _1\le \theta _2$ and $\theta _2\le \theta _1$ is not valid. Suppose that $\theta _1\le \theta _2$ is *not* satisfied. Since $\theta _1\gg 0=0\cdot \theta _2$, the set $\{\lambda \ge 0: \theta _1\ge \lambda \cdot \theta _2\}$ is non-empty and the following supremum is well-defined

$$\begin{aligned} \lambda _0=\sup \{\lambda \ge 0: \theta _1\ge \lambda \theta _2\}. \end{aligned}$$

Since $\theta _1\gg 0$ there exists $\epsilon >0$ such that $\theta _1\ge \epsilon \theta _2$, hence $\lambda _0\ge \epsilon >0$. On the other hand, by the assumption $\theta _1\not \le \theta _2$, therefore we also have $1\not \in \{\lambda \ge 0: \theta _1\ge \lambda \theta _2\}$, thus $\lambda _0\i {\mathbf {n}}(0,1)$. By the continuity, $\theta _1\ge \lambda _0 \theta _2,$ by the monotonicity of $\bar{{\mathcal {K}}}$ and $\lambda _0<1$ one has

$$\begin{aligned} \theta _1&=\bar{{\mathcal {K}}}\theta _1 \ge \bar{{\mathcal {K}}}(\lambda _0 \theta _2) =\lambda _0{\mathscr {R}}_{\lambda _0}(\theta _2)\gg \lambda _0{\mathscr {R}}_{1}(\theta _2)\\&=\lambda _0\bar{{\mathcal {K}}}\theta _2=\lambda _0 \theta _2, \end{aligned}$$

Thus, $\theta _1\gg \lambda _0 \theta _2$, implying $\theta _1\ge (\delta +\lambda _0) \theta _2$ for some small positive $\delta$. The latter inequality contradicts the definition of $\lambda _0$. This finishes the proof of the uniqueness. $\square$

### Asymptotic behaviour of a general solution of (61)

Let us return to the general Eq. (61). If the initial distribution of population vanishes: ${\mathbf {n}}(a,0)={\mathbf {f}}(a)=0$, the uniqueness of solution of (58) immediately implies that the population density ${\mathbf {n}}(a,t)\equiv 0$ for all $a,t\ge 0$. This conclusion also holds true even under a weaker assumption that ${\mathcal {F}}{\mathbf {f}}\equiv 0$. The latter is evident from the biological point of view: the population disappears if its initial distribution is older that the maternity period. Taking into account these observations, it is naturally to assume that

81 $$\begin{aligned} {\mathcal {F}}{\mathbf {f}}\not \equiv 0. \end{aligned}$$

The main result of this section states that under this assumption, any solution of (61) behaves asymptotically as the maximal solution.

#### Theorem 2

Let $\chi$ be the solution to (61) satisfying (81). Then

82 $$\begin{aligned} \lim _{t\rightarrow \infty }\chi (t)=\theta . \end{aligned}$$

We start with two results describing the upper and lower solutions to Eq. (61).

#### Lemma 12

Let $\chi$ be a solution to (61). Then

83 $$\begin{aligned} \limsup _{t\rightarrow \infty }\chi (t)\le \theta , \end{aligned}$$

where the latter inequality should be understood component-wise.

#### Proof

Let $\rho ^+$ be an arbitrary stationary upper solution to (61), i.e.

84 $$\begin{aligned} \rho ^+\ge {\mathscr {L}}_{\mathbf {f}}\rho ^+. \end{aligned}$$

Notice that that the class of stationary upper solutions is nonempty. Indeed, it follows from (53) that, for example, $2(\omega _2 +\epsilon ){\mathbf {1}}$ is such a an upper solution for any $\epsilon >0$. Now, let us define the iterative sequence by

$$\begin{aligned} \rho ^{(i)}&=\bar{{\mathcal {K}}}^{i+1} \rho ^+\quad \text {for}\quad i\ge 0\quad \text {and}\quad \rho ^{(0)}=\rho ^+,\\ \chi ^{(i)}&={\mathscr {L}}_{\mathbf {f}}^{i+1} \rho ^+ \quad \text {for}\quad i\ge 0\quad \text {and}\quad \chi ^{(0)}=\rho ^+. \end{aligned}$$

Then applying the argument of the proof of Proposition 9 yields that $\{\rho ^{(i)}\}$ is non-increasing:

$$\begin{aligned} \rho ^{(i+1)}\le \rho ^{(i)}, \quad \forall i\ge 0. \end{aligned}$$

Also, since $\rho ^{(i)}$ is a constant vector function, it follows by Lemma 8 that ${\mathscr {L}}_{\mathbf {f}} \rho ^{(i)}\in S_{A_m}$ and also that

$$\begin{aligned} {\mathscr {L}}_{\mathbf {f}} \rho ^{(i)}(t) \equiv \bar{{\mathcal {K}}}\rho ^{(i)}(t),\quad \forall t\ge A_m. \end{aligned}$$

We claim that for any $j\ge 0$

- $$\chi ^{(j+1)}\le \chi ^{(j)}$$ for all $$t\ge 0$$;
- $$\chi ^{(j)}= \rho ^{(j)}$$ for $$t\ge jA_m$$.

The proof is by induction. Notice that (b) holds trivially for $j=0$, and by the assumption (84)

$$\begin{aligned} \chi ^{(1)}={\mathscr {L}}_{\mathbf {f}}\chi ^{(0)}= {\mathscr {L}}_{\mathbf {f}}\rho ^{+}\le \rho ^+=\chi ^{(0)} \end{aligned}$$

which yields (a) for $j=0$. Let the claims (a)–(b) hold true for some $j\ge 1$. Then (a) follows from the monotonicity of ${\mathscr {L}}_{\mathbf {f}}$:

$$\begin{aligned} \chi ^{(j+1)}={\mathscr {L}}_{\mathbf {f}}\chi ^{(j)}\le {\mathscr {L}}_{\mathbf {f}}\chi ^{(j-1)}=\chi ^{(j)}. \end{aligned}$$

Furthermore by the assumption $\chi ^{(j)}\in S_{jA_m}$ and $\chi ^{(j)}_\infty = \rho ^{(j)}$. Hence Lemma 8 yields

$$\begin{aligned} \chi ^{(j+1)}={\mathscr {L}}_{\mathbf {f}}\chi ^{(j)}\in S_{(j+1)A_m} \end{aligned}$$

and

$$\begin{aligned} \chi ^{(j+1)}_\infty =({\mathscr {L}}_{\mathbf {f}}\chi ^{(j)})_\infty =\bar{{\mathcal {K}}}\chi ^{(j)}_\infty = \bar{{\mathcal {K}}}\rho ^{(j)}=\rho ^{(j+1)}, \end{aligned}$$

which yields (b) for $j+1$.

Next, it follows from (a) and the boundedness of the image of ${\mathcal {L}}$ that $\{\chi ^{(j)}\}$ is non-increasing and bounded from below, thus has a limit which obviously is a solution of (61). By the uniqueness, $\lim _{j\rightarrow \infty }\chi ^{(j)}(t)=\chi (t)$. Now, let $1\le k\le N$. Then the sequence of the coordinate functions $\chi _k^{(j)}(t)$ is non-increasing with respect to *j* and $\lim _{j\rightarrow \infty }\chi _k^{(j)}(t)=\chi _k(t)$. Let $\epsilon >0$. Since $\lim _{j\rightarrow \infty }\rho _k^{(j)}=\theta _k$, there exists $j_0$ such that $\theta _k\le \rho _k^{(j)}\le \theta _k+\epsilon$ for all $j\ge j_0$. This implies that $\chi _k^{(j)}(t)\le \theta _k+\epsilon$ for all $j\ge j_0$ and $t\ge jA_m$. Passing to the limit $j\rightarrow \infty$ we obtain $\chi _k(t)\le \theta _k+\epsilon$ for $t\ge jA_m$ which easily implies (83). $\square$

#### Lemma 13

Let $\chi$ be a solution to (61). If there exists a lower solution $\rho ^-$ to (61), i.e. ${\mathscr {L}}_{\mathbf {f}}\rho ^-\ge \rho ^-$ such that $\rho ^-\in S_M$ for some $M\ge 0$ and $\rho ^-_\infty \ne 0$ then $\lim _{t\rightarrow \infty }\chi (t)=\theta$.

#### Proof

As above, let us consider the sequence of iterations

$$\begin{aligned} \chi ^{(j)}&={\mathscr {L}}_{\mathbf {f}}^j\chi ^{(0)}\quad \text {for}\quad i\ge 0 \quad \text {and}\quad \chi ^{(0)}(t)=\rho ^{-},\\ \rho ^{(j)}&=\bar{{\mathcal {K}}}^j\rho ^{(0)} \quad \text {for}\quad i\ge 0\quad \text {and}\quad \rho ^{(0)}(t)=(\rho ^{-})_\infty , \end{aligned}$$

By Lemma 8, $\chi ^{(j)}\in S_{M+jA_m}$. Furthermore, by (68)

$$\begin{aligned} \chi ^{(1)}_\infty =({\mathscr {L}}_{\mathbf {f}}\rho ^-)_\infty =\bar{{\mathcal {K}}}\rho ^{-}_\infty =\rho ^{(1)}. \end{aligned}$$

Using an induction argument readily yields

85 $$\begin{aligned} \chi ^{(j)}_\infty =\rho ^{(j)}, \quad \forall j\ge 0. \end{aligned}$$

Since ${\mathscr {L}}_{\mathbf {f}}\rho ^-\ge \rho ^-$, we have $\chi ^{(1)}\ge \chi ^{(0)}$, thus by the monotonicity of ${\mathscr {L}}_{\mathbf {f}}$, $\chi ^{(j+1)}\ge \chi ^{(j)}$. This proves that $\{\chi ^{(j)}(t)\}$ is a nondecreasing sequence. Furthermore, (85) implies that

$$\begin{aligned} \rho ^{(j+1)}=\chi ^{(j+1)}_\infty \ge \chi ^{(j)}_\infty =\rho ^{(j)}, \end{aligned}$$

thus, $\{\rho ^{(j)}\}$ is also a nondecreasing sequence. Furthermore, since $\rho ^-_\infty \ne 0$, we have that $\rho ^{(j)}\gg 0$ for $j\ge 1$. By Lemma 6 the both sequences are bounded from above by $\omega _2 {\mathbf {1}}_N$. Thus, the limits $\rho :=\lim _{j\rightarrow \infty }\rho ^{(j)}$ and ${{\bar{\chi }}}:=\lim _{j\rightarrow \infty }\chi ^{(j)}(t)$ exist and solve $\bar{{\mathcal {K}}}\rho =\rho$ and ${\mathscr {L}}_{\mathbf {f}}{{\bar{\chi }}}={{\bar{\chi }}}$, respectively, where $\rho \gg 0$. By the corresponding uniqueness results, we have $\rho =\theta$ and ${{\bar{\chi }}}=\chi$. Arguing as in Lemma 12, we obtain $\liminf _{t\rightarrow \infty }\chi (t)\ge \theta$ (the latter is understood component-wise). Hence (83) implies the existence of the limit $\lim _{t\rightarrow \infty }\chi (t)=\theta$. $\square$

#### Proof of Theorem 2

If $R_0\le 1$, then Theorem 1 yields $\theta \equiv 0$, then (83) immediately yields (82). Therefore we shall suppose that $R_0>1$. Let $\chi$ be the unique solution to (61) and let $\theta \gg 0$ be the unique maximal solution of (67). By Lemma 13, it suffices to show that there exists a lower solution $\rho ^-$ to (60) such that $\rho ^-\in S_M$ for some $M\ge 0$ and $\rho ^-_\infty \ne 0$. In the remained part of the proof we shall construct such a solution. Let us consider an auxiliary sequence of iterations

$$\begin{aligned} \rho ^{(j)}={\mathscr {L}}_{\mathbf {f}}\rho ^{(0)}\quad \text {for}\quad j\ge 1 \quad \text {and}\quad \rho ^{(0)}\equiv 0. \end{aligned}$$

We claim that the new function $\rho ^-(t)$ defined by

86 $$\begin{aligned} \rho ^-(t)= \left\{ \begin{array}{ll} \rho ^{(j)}(t), &{}\quad 0\le t\le M, \\ \lambda \theta , &{}\quad t>M, \end{array} \right. \end{aligned}$$

is a lower solution to Eq. (61) for certain $M>A_m$, sufficiently large $j\ge 1$ and sufficiently small $\lambda >0$ to be specified later. To this end, first notice that

$$\begin{aligned} \rho ^{(1)}=\varphi :={\mathcal {F}} {\mathbf {f}} \ge 0=\rho ^{(0)}, \end{aligned}$$

hence using an induction by $j\ge 1$, one gets

$$\begin{aligned} \rho ^{(j+1)}={\mathscr {L}}_{\mathbf {f}}\rho ^{(j)} \ge {\mathscr {L}}_{\mathbf {f}}\rho ^{(j-1)} =\rho ^{(j)}, \end{aligned}$$

i.e. the sequence $\rho ^{(j)}$ is non-decreasing in *j*. It also follows from the latter inequality that $\rho ^{(j)}\le {\mathscr {L}}_{\mathbf {f}}\rho ^{(j)}$, i.e. $\rho ^{(j)}$ is a lower solution to (61). Hence, $\rho ^{-}(t)$ defined by (86) is a lower solution to (60) in the interval $t\in [0,M]$. In particular,

$$\begin{aligned} ({\mathscr {L}}_{\mathbf {f}}\rho ^-)(t)-\rho ^-(t)\ge 0\quad \text {for}\quad t\in [0,M]. \end{aligned}$$

Next, we assume that $t\in [M,M+A_m]$. By the assumption $M>A_m$, hence one has $({\mathcal {F}}{\mathbf {f}})(t)=0$ and ${\mathscr {L}}_{\mathbf {f}}\rho ^-={\mathcal {K}}\rho ^-$. We have by (86) and condition (H4) that

$$\begin{aligned} {\mathcal {K}}\rho ^-(t)&= \int _{0}^{A_m}{\mathbf {m}}(a)\varPhi (a;\rho ^{-},t-a)\,da \\&= \int _{0}^{t-M}{\mathbf {m}}(a)\varphi (a;\lambda \theta )\,da + \int _{t-M}^{A_m}{\mathbf {m}}(a)\varPhi (a;\rho ^{(j)},t-a)\,da \end{aligned}$$

On the other hand, since $\bar{{\mathcal {K}}}\theta =\theta$, we have

$$\begin{aligned} \theta = \int _0^{A_m}{\mathbf {m}}(a)\varphi (a;\theta )\,da. \end{aligned}$$

This yields by virtue of $\rho ^-(t)=\lambda \theta$ for $t\in (M,M+A_m)$ and (65) that

87 $$\begin{aligned} ({\mathscr {L}}_{\mathbf {f}}\rho ^--\rho ^-)(t)&=({\mathcal {K}}\rho ^--\rho ^-)(t)=({\mathcal {K}}\rho ^--\theta )(t) \nonumber \\&= \int _{0}^{t-M}{\mathbf {m}}(a)(\varphi (a;\lambda \theta )-\lambda \varphi (a;\theta ))\,da \end{aligned}$$

88 $$\begin{aligned}&\quad + \int _{t-M}^{A_m}{\mathbf {m}}(a)(\varPhi (a;\rho ^{(j)},t-a) -\lambda \varPhi (a;\theta ,t-a ))\,da. \end{aligned}$$

We claim that the integrals (87) and (88) are nonnegative. The first integral is nonnegative by virtue of Corollary 5. To show that (88) is nonnegative, let us estimate function $\varPhi _k(a;\rho ^{(j)},t-a)$ from below. By (H6), $m_k(a)\ge \delta >0$ for all $a\in [a_k,b_k]$, where $a_k\ge \beta _k$ and $b_k$ are the same as in Lemma 7. Since ${{\mathcal {F}}}({\mathbf {f}})$ is not identically zero, there exists an interval $[s_1,s-2]$, where this function is positive. Applying Lemma 7 for $\rho =\rho ^{(1)}={\mathcal F}({\mathbf {f}})$, we get that

$$\begin{aligned} ({{\mathcal {K}}}\rho ^{(1)})_k(t)>0\quad \text{ for }\quad t\in [s_1+a_k,s_2+b_k]. \end{aligned}$$

Therefore

$$\begin{aligned} \rho ^{(2)}(t)={{\mathcal {K}}}\rho ^{(1)}(t)+{\mathcal F}{\mathbf {f}}(t)>0\quad \text{ for }\quad t\in [s_1+a_k,s_2+b_k], k=1,\ldots ,N, \end{aligned}$$

and, in particular this is true for $k=1$. Repeating this argument yields

$$\begin{aligned} \rho ^{(j)}(t)>0\quad \text{ for }\quad t\in [s_1+(j-1)a_1,s_2+(j-1)b_1]. \end{aligned}$$

This implies that

$$\begin{aligned} \varPhi _k(a;\rho ^{(j)},t-a)>0\quad \text{ for }\quad a\ge \beta _k\quad \text{ and }\quad t-a\in [s_1+(j-1)a_1,s_2+(j-1)b_1]. \end{aligned}$$

Now we choose the index *j* and the number *M* to satisfy

$$\begin{aligned}{}[M-A_m,M+A_m]\subset [s_1+(j-1)a_1,s_2+(j-1)b_1]. \end{aligned}$$

Then

89 $$\begin{aligned} \varPhi _k(a;\rho ^{(j)},t-a)>0\quad \text{ for }\quad a\in [\beta _k,A_m]\quad \text{ and }\quad t\in [0,A_m]. \end{aligned}$$

Therefore,

$$\begin{aligned} \varPhi _k(a;\rho ^{(j)},t-a)\le \lambda \varPhi _k(a;\theta ,t-a) \end{aligned}$$

for such *a* and *t* if $\lambda$ is sufficiently small positive number. This gives positivity of (88) for $t\ge M+a_k$. If $t\le M+a_k$ then the first integral in (88) is estimated from below by

$$\begin{aligned} \int _{a_k}^{b_k}m_k(a)\varPhi _k(a;\rho ^{(j)},t-a)da \end{aligned}$$

and it is positive for $t\in [M,M+A_m]$. Since the functions $\varPhi _k$ are uniformly bounded this implies the positivity of (88) for $M\le t\le M+a_k$ when $\lambda$ is small.

Finally, if $t\ge M+A_m$, then since ${\mathcal {F}}{\mathbf {f}}(t)=0$ we have by virtue of Corollary 5 that

$$\begin{aligned} ({\mathscr {L}}_{\mathbf {f}}\rho ^--\rho ^-)_k(t)=({\mathcal {K}}\rho ^--\rho ^-)_k(t) = \int _{0}^{A_m}m_k(a)(\varphi _k(a;\lambda \theta ) -\lambda \varphi _k(a;\theta ))\,da \ge 0 \end{aligned}$$

This proves that the function $\rho ^-(t)$ defined by (86) is a lower solution to Eq. (67), therefore by Lemma 13 we have the desired convergence that completes the proof. $\square$

### Asymptotics of total population

According to the assumption made in the beginning of this section, the maximal length of life is constant: $B(t)\equiv b$. Then the total (multipatch) population ${\mathbf {P}}(t)$ at time *t* is the vector-function

90 $$\begin{aligned} {\mathbf {P}}(t)=\int _0^{b}{\mathbf {n}}(a,t)\,da. \end{aligned}$$

Then we have the following result.

#### Theorem 3

Let *n*(*a*, *t*) be the solution of (58) and let the condition (81) hold. Then the following dichotomy holds: if $R_0\le 1$ then ${\mathbf {P}}(t)\rightarrow 0$ as $t\rightarrow \infty$, and if $R_0>1$ then

91 $$\begin{aligned} \lim _{t\rightarrow \infty }{\mathbf {P}}(t)= \int _0^{b}\varphi (a;\theta )\,da, \end{aligned}$$

where $\theta$ is the maximal solution to the characteristic equation.

#### Proof

Denote by $\rho (a)$ the newborns function determined by ${\mathbf {f}}(a)$ by virtue of (61). We have for general $t>0$

$$\begin{aligned} {\mathbf {P}}(t)= \int _0^{\min \{t,b\}}\varPhi (a;\rho ,t-a)\,da+\int _{\min \{t,b\}}^{b}\varPsi (a;{\mathbf {f}},a-t)da. \end{aligned}$$

On the other hand, by (H5) ${{\,\mathrm{\mathrm {supp}}\,}}{\mathbf {f}}\subset [0,b]$, hence using (59) we have for any $t>b$ that

$$\begin{aligned} {\mathbf {P}}(t)= \int _0^{b}\varPhi (a;\rho ,t-a)\,da. \end{aligned}$$

Next, by Theorem 1 and Theorem 2 we have $\lim _{t\rightarrow \infty }\rho (t)=\theta$ and furthermore by (40) there holds $h(a):=\varPhi (a;\rho ,t-a)$ satisfies

92 $$\begin{aligned} \left\{ \begin{aligned} \frac{d }{da}{h}(a)&=-{\mathbf {M}}({h}(a),a){h}(a)+{\mathbf {D}}(a){h}(a), \\ h(0)&= \rho (t-a), \end{aligned} \right. \end{aligned}$$

By continuity of solutions (92) with respect to a parameter and (64), we have for any fixed $a>0$ that

$$\begin{aligned} \lim _{t\rightarrow \infty }\varPhi (a;\rho ,t-a)=\varphi (a;\theta ). \end{aligned}$$

This readily yields (91). $\square$

### Estimates for the basic reproduction number and for the maximal solution

In this section we shall assume that the condition (12) hold, i.e.

$$\begin{aligned} \sum _{k=1}^ND_{kj}(a)\le 0, \quad 1\le j\le N. \end{aligned}$$

The biological meaning of the latter inequality is that individuals do not reproduce during migration (but can die). This condition immediately implies that

$$\begin{aligned} D_{kk}(a)\le 0. \end{aligned}$$

Throughout this section, we use the following notation:

$$\begin{aligned} m(a)=\max _{1\le k\le N}m_k(a),\quad \mu (a):=\min _{1\le k\le N}\mu _k(a). \end{aligned}$$

#### Proposition 10

Under the made assumptions,

93 $$\begin{aligned} \max _{1\le k\le N}\int _0^{\infty }\!\!m_k(a)e^{-\int _0^a(\mu _k(v)+|D_{kk}(v)|)dv}\,da \le R_0\le \int _0^{\infty }\!\!m(a)e^{-\int _0^a\mu (v)dv}\,da. \end{aligned}$$

#### Proof

By Corollary 7 there exists an eigenvector $\rho \gg 0$ of ${\mathscr {R}}_0$ corresponding the maximal eigenvalue $R_0$, i.e. ${\mathscr {R}}_0\rho =R_0\rho$. Let us consider the problem (74) with the initial condition $x=\rho$. Using the assumption (12) and summing up the Eq. (74) for all $1\le k\le N$ we obtain that $\psi (a)=\sum _{k=1}^N Y_k(a;\rho )$ satisfies

$$\begin{aligned} \left\{ \begin{aligned} \frac{d }{da}\sum _{k=1}^N\psi (a)&\le -\mu (a)\psi (a),\\ \psi (0)&=\sum _{k=1}^N\rho _k, \end{aligned} \right. \end{aligned}$$

which readily yields

$$\begin{aligned} \psi (a)\le e^{-\int _o^a\mu (v)dv}\sum _{k=1}^N\rho _k. \end{aligned}$$

Then by (75)

$$\begin{aligned} R_0\sum _{k=1}^N\rho _k=\sum _{k=1}^N({\mathscr {R}}_{0}x)_k\le \int _0^{\infty }m(a)\psi (a)\,da\le \sum _{k=1}^N\rho _k\int _0^{\infty }m(a)e^{-\int _o^a\mu (v)dv}\,da. \end{aligned}$$

Since the sum $\sum _{k=1}^N\rho _k>0$ we arrive at the right hand side of (93).

Now, in order to prove the left hand side inequality in (93), notice that in the made notation by virtue of $D_{kj}(a)\ge 0$ for $k\ne j$ and $Y_j(a,\rho )\ge 0$ for all admissible *a* we have

$$\begin{aligned} \frac{d}{da}Y_k(a;\rho ) \ge D_{kk}(a)Y_k(a;\rho )=-(\mu _k(a)+|D_{kk}(a)|)Y_k(a;\rho ), \end{aligned}$$

which yields in virtue of $Y_k(0,\rho )=\rho _k$ that

$$\begin{aligned} Y_k(a;\rho )\ge \rho _ke^{-\int _0^a(\mu _k(v)+|D_{kk}(v)|)dv}. \end{aligned}$$

Combining this with (75) we obtain

$$\begin{aligned} R_0\rho _k =\int _0^{\infty }m_k(a)Y_k(a;\rho )\,da \ge \rho _k \int _0^{\infty }m_k(a)e^{-\int _0^a(\mu _k(v)+|D_{kk}(v)|)dv}\,da, \end{aligned}$$

thus implying (93) by virtue of $\rho _k>0$. $\square$

#### Remark 1

The estimates (93) are optimal. Indeed, if $D_{kj}\equiv 0$, the system (74) splits into separate equations

$$\begin{aligned} \frac{d}{da}Y_k(a;x)=-(\mu _k(a)+|D_{kk}(a)|)Y_k(a;x), \quad Y_k(0,x)=x_k, \quad 1\le k\le N, \end{aligned}$$

implying that each $e_k$ is an eigenvector of ${\mathscr {R}}_0$ with eigenvalue

$$\begin{aligned} \lambda _k=\int _{0}^\infty m_k(a)e^{-\int _{0}^a(\mu _k(a)+|D_{kk}(a)|)ds}da, \end{aligned}$$

therefore $R_0=\max _{k}\lambda _k$ is exactly the left hand side of (93). On the other hand, suppose all patches to have the same birth and death rates: $m_k(a)\equiv m(a)$ and $\mu _k(a)\equiv \mu (a)$ for any $1\le k\le N$, and also that the dispersion is absent: $D\equiv 0$. Then a similar argument yields $R_0=\int _0^{\infty }\!\!m(a)e^{-\int _0^a\mu (v)dv}\,da$ implying the exactness of the upper estimate in (93).

In order to establish the corresponding estimates for the maximal solution $\theta$ we consider an auxiliary function

94 $$\begin{aligned} {\widetilde{M}}(t,a):=\frac{1}{t}\min _{\xi \in S(t)}\sum _{i=1}^N \xi _iM_i(\xi _i,a),\quad t>0, \end{aligned}$$

where the minimum is taken over the simplex

$$\begin{aligned} S(t)=\{\xi \in {\mathbb {R}}^{N}_+: \sum _{i=1}^N\xi _i=t\}. \end{aligned}$$

#### Lemma 14

In the above notation, ${\widetilde{M}}(t,a)$ is nondecreasing in $t>0$ and

95 $$\begin{aligned} \lim _{t\rightarrow +0}{\widetilde{M}}(t,a)=\mu (a). \end{aligned}$$

Furthermore,

96 $$\begin{aligned} {\widetilde{M}}(t,a)-\mu (a)\ge \frac{p(a)}{N^\gamma } t^\gamma , \end{aligned}$$

where *p*(*a*) is the function from (H2).

#### Proof

If $0<t'\le t$ then $\lambda =t'/t\le 1$. If $\xi \in S(t)$ is the minimum point of (94) then $\xi '=\lambda \xi \in S(t')$, hence using the monotonicity condition in (H2) and $\xi \ge \xi '$ we obtain

$$\begin{aligned} {\widetilde{M}}(t,a)=\frac{1}{t}\sum _{i=1}^N \xi _iM_i(\xi _i,a)= \frac{1}{\lambda t}\sum _{i=1}^N \xi '_iM_i(\xi _i,a)\ge \frac{1}{\lambda t}\sum _{i=1}^N \xi '_iM_i(\xi '_i,a)\ge {\widetilde{M}}(t',a). \end{aligned}$$

which yields the nondecreasing monotonicity. In particular the limit in (95) does exist. Denote it by ${\widetilde{\mu }}$. Since $M_k(\xi _k,a)\ge \mu _k(a)\ge \mu (a)$, we have ${\widetilde{M}}(t,a)\ge \mu (a)$. In particular, ${\widetilde{\mu }}\ge \mu (a)$. Conversely, given $t>0$ let $\xi \in S(t)$ be the corresponding minimum point of (94). Let the number $k=k(a)$, $1\le k\le N$, be chosen such that $\mu (a)=M_k(0,a)$. Define $\eta _i=0$ for $i\ne k$ and $\eta _k=t$. Then

$$\begin{aligned} {\widetilde{M}}(t,a)=\frac{1}{t}\sum _{i=1}^N \xi _iM_i(\xi _i,a)\le \frac{1}{t}\sum _{i=1}^N \eta _iM_i(t,a)=M_k(t,a). \end{aligned}$$

Passing to the limit as $t\rightarrow +0$ in the latter inequality yields ${\widetilde{\mu }}\le \mu (a)$, thus implying (95).

Finally, assume again that $\xi \in S(t)$ is the minimum point of (94) for $t>0$. Then using (H2) and the Hölder inequality we obtain

$$\begin{aligned} t({\widetilde{M}}(t,a)-\mu (a))&=\sum _{i=1}^N(M_i(\xi _i,a)-\mu (a)) \xi _i\ge \sum _{i=1}^N(M_i(\xi _i,a)-M_i(0,a)) \xi _i\\&\ge p(a)\sum _{k=1}^N\xi _i^{1+\gamma }\ge \frac{p(a)}{N^\gamma }\left( \sum _{i=1}^N\xi _i\right) ^{1+\gamma } =\frac{p(a)}{N^\gamma }t^{1+\gamma }, \end{aligned}$$

which yields (96). $$\square $$

#### Proposition 11

In the notation of Proposition 10, if $R_0>1$ then there exists a unique $\theta _+>0$ such that

97 $$\begin{aligned} \int _0^{\infty } \frac{m(a)\,e^{-\int _0^a\mu (s)\,ds}}{(1+\theta _+^\gamma P(a))^{1/\gamma }}= 1, \end{aligned}$$

where

$$\begin{aligned} P(a)=\frac{\gamma }{N^\gamma } \int _{0}^{a}p(t)e^{-\int _0^t \mu (s)ds}dt. \end{aligned}$$

Furthermore,

98 $$\begin{aligned} \sum _{k=1}^N\theta _k \le \theta _+. \end{aligned}$$

#### Proof

Since $R_0>1$, the maximal solution $\theta \gg 0$ and $\theta =\bar{{\mathcal {K}}}\theta$. Let $\varphi _k(a,\theta )$ denote the corresponding solution of (64) satisfying (63). Let $\psi (a)=\sum _{k=1}^N\varphi _k(a;\theta )$. Then summing up Eq. (64) and using (12) and (94) we obtain

$$\begin{aligned} \frac{d }{da}\psi (a)\le -\sum _{k=1}^NM_k(\varphi _k(a;\theta ),a) \varphi _k(a;\theta ) \le -{\widetilde{M}}(\psi (a),a)\psi (a), \end{aligned}$$

The obtained inequality implies that $\psi (a)$ is a (positive) decreasing function of $a\ge 0$, in particular, $0<\psi (a)\le \psi (0)=\Vert \theta \Vert _{\infty }$. We have from (96)

$$\begin{aligned} \frac{d}{da}\psi (a)+\mu (a)\psi (a)\le -({\widetilde{M}}(\psi (a),a)-\mu (a))\psi (a) \le -\frac{p(a)}{N^\gamma }\psi (a)^{1+\gamma }. \end{aligned}$$

Rewriting the obtained inequality for $z(a)=\psi (a)\exp (\int _0^a\mu (s)\,ds)$as

$$\begin{aligned} \frac{dz(a)}{da}\le -\frac{p(a)}{N^\gamma }z(a)^{1+\gamma }e^{-\gamma \int _0^a\mu (s)\,ds}, \end{aligned}$$

yields after integrating

$$\begin{aligned} \frac{1}{z(a)^\gamma }-\frac{1}{z(0)^\gamma }\ge \frac{\gamma }{N^\gamma } \int _{0}^{a}p(t)e^{-\gamma \int _0^t \mu (s)ds}dt=P(a) \end{aligned}$$

This yields by virtue of $z(0)=\psi (0)=\Vert \theta \Vert _{\infty }$

99 $$\begin{aligned} \psi (a)\le \frac{\Vert \theta \Vert _{\infty }\,e^{-\int _0^a\mu (s)\,ds}}{(1+P(a)\Vert \theta \Vert _{\infty }^\gamma )^{1/\gamma }} \end{aligned}$$

Next, since $\theta =\bar{{\mathcal {K}}}\theta$, it readily follows that

$$\begin{aligned} \Vert \theta \Vert _{\infty }\le \int _0^{\infty }m(a)\psi (a)\,da \le \Vert \theta \Vert _{\infty } \int _0^{\infty } \frac{m(a)\,e^{-\int _0^a\mu (s)\,ds}}{(1+ P(a)\Vert \theta \Vert _{\infty }^\gamma )^{1/\gamma }}. \end{aligned}$$

This yields by virtue of $\Vert \theta \Vert _{\infty }>0$ that

$$\begin{aligned} \int _0^{\infty } \frac{m(a) \,e^{-\int _0^a\mu (s)\,ds}}{(1+P(a)\Vert \theta \Vert _{\infty }^\gamma )^{1/\gamma }}\ge 1. \end{aligned}$$

Since the integral

$$\begin{aligned} I(t)=\int _0^{\infty }\frac{m(a) \,e^{-\int _0^a\mu (s)\,ds}}{(1+P(a)t^\gamma )^{1/\gamma }} \end{aligned}$$

is a decreasing function of *t* and $\lim _{t\rightarrow \infty }I(t)=0$, there exists (a unique) $\theta _+\ge \Vert \theta \Vert _{\infty }$ solving the Eq. (97), thereby proving (98). $\square$

#### Remark 2

Let us comment on (98) from the biological point of view. Notice by Theorem 2 that $\sum _{k=1}^N\theta _k$ is the asymptotical value of the total number of newborns on all patches. By the dichotomy, $R_0\le 1$ implies $\theta =0$, thus the total asymptotical number of newborns is zero. On the other hand, in the nontrivial case $R_0>1$, hence by (93) $\int _0^{\infty }m(a)e^{-\int _0^a\mu (v)dv}\,da>1$, which easily implies that (97) has a positive solution.

The next proposition provides a lower estimate for the maximal solution.

#### Proposition 12

Let there exist a function $q(a)>0$ such that

100 $$\begin{aligned} M_k(v,a)-M_k(0,a)\le q(a) v^{\gamma }, \quad \forall (v,a)\in {\mathbb {R}}^{2}_+. \end{aligned}$$

If for some *k*

101 $$\begin{aligned} \int _0^{\infty }m_k(a)e^{-\int _0^a(\mu _k(v)+|D_{kk}(v)|)dv}\,da>1 \end{aligned}$$

then

102 $$\begin{aligned} \theta ^-_k \le \theta _k, \end{aligned}$$

where $\theta ^-_k$ is the unique solution to equation

103 $$\begin{aligned} \int _0^{\infty }\frac{m_k(a)e^{-\gamma \int _0^a(\mu (s))+|D_{kk}(a)|)\,ds}}{(1+(\theta ^-_k)^\gamma Q(a))^{1/\gamma }}\,da=1, \end{aligned}$$

and

$$\begin{aligned} Q(a)=\frac{\gamma }{N^\gamma } \int _{0}^{a}q(t)e^{-\int _0^t (\mu (s)+|D_{kk}(s)|)ds}dt. \end{aligned}$$

#### Proof

First notice that (101) implies by (93) that $R_0>1$, thus $\theta \gg 0$. Since $D_{kj}(a)\ge 0$ for $j\ne k$, the *k*-th equation in (64) yields

$$\begin{aligned} \frac{d}{da}\varphi _k(a;\theta ) \ge -(M_k(\varphi _k(a,\theta ),a)-D_{kk}(a))\varphi _k(a;\theta ), \end{aligned}$$

hence using (100) we obtain by virtue of $M_k(0,a)=\mu _k(a)$ that

$$\begin{aligned} \frac{d}{da}\varphi _k(a;\theta )+(\mu _k(a)-D_{kk}(a))\varphi _k(a)\ge -q(a)\varphi _k(a)^{1+\gamma }. \end{aligned}$$

Arguing similar to the proof of Proposition 11 we get from $\varphi _k(0;\theta )=\theta _k$ that

104 $$\begin{aligned} \varphi _k(a;\theta )\ge \theta _k\frac{\,e^{-\int _0^a(\mu (s)+|D_{kk}(a)|)\,ds}}{(1+\theta _k^\gamma Q(a))^{1/\gamma }}, \end{aligned}$$

therefore

$$\begin{aligned} \theta _k=(\bar{{\mathcal {K}}}(\theta ))_k \ge \theta _k \int _0^{\infty }\frac{m_k(a)e^{-\gamma \int _0^a(\mu (s))+|D_{kk}(a)|)\,ds}}{(1+\theta _k^\gamma Q(a))^{1/\gamma }}\,da. \end{aligned}$$

Since $\theta \gg 0$, one has $\theta _k>0$, hence

$$\begin{aligned} \int _0^{\infty }\frac{m_k(a)e^{-\gamma \int _0^a(\mu (s))+|D_{kk}(a)|)\,ds}}{(1+\theta _k^\gamma Q(a))^{1/\gamma }}\,da\le 1. \end{aligned}$$

Again, let

$$\begin{aligned} I(t)=\int _0^{\infty }\frac{m_k(a)e^{-\gamma \int _0^a(\mu (s))+|D_{kk}(a)|)\,ds}}{(1+\theta _k^\gamma Q(a))^{1/\gamma }}\,da. \end{aligned}$$

Then *I*(*t*) is decreasing, $I(\theta _k)\le 1$ and by (101) $I(0)>1$, thus there exists (a unique) solution $\theta ^{-}_k$ of (103) such that $\theta _k\ge \theta ^{-}_k$. $\square$

## Periodically varying environment

We consider an important particular case of the main problem (1)–(4) when the environment is periodically changing. In this section and in the rest of the paper, it is assumed that the vital rates, regulating function and dispersion coefficients are time-dependent and periodic with a period $T>0$. The boundary-initial value problem (1)–(4) is now in a *T*-periodic domain ${\mathscr {B}}$, where $B(t+T)=B(t)$, $t\in {\mathbb {R}}^{}$, under the periodicity assumption that

105 $$\begin{aligned} \begin{aligned} {\mathbf {m}}(a,t+T)&= {\mathbf {m}}(a,t), \\ {\mathbf {M}}(v,a,t+T)&= {\mathbf {M}}(v,a,t),\\ {\mathbf {D}}(a,t+T)&= {\mathbf {D}}(a,t) \end{aligned}\end{aligned}$$

for any $1\le k,j\le N$. Throughout this section, we assume that the conditions (H1)–(H5) are satisfied.

Notice that the existence and uniqueness of a solution ${\mathbf {n}}(a,t)$ to the periodic problem follows from the general result given by Proposition 6 and it is given explicitly by (46). Note also that ${\mathbf {n}}(a,t)$ need not to be periodic in *t* but it is natural to expect that ${\mathbf {n}}(a,t)$ converges to a *T*-periodic function $\rho (t)$ for *t* sufficient large, where $\rho (t)$ solves the associated *characteristic equation*

106 $$\begin{aligned} \widetilde{{\mathcal {K}}}\rho (t)=\rho (t), \quad t\in {\mathbb {R}}. \end{aligned}$$

Here the operator $\widetilde{{\mathcal {K}}}$ is defined by

$$\begin{aligned} \widetilde{{\mathcal {K}}}\rho (t):=\int _0^{\infty }{\mathbf {m}}(a,t)\varPhi (a;\rho ,t-a)\,da, \quad t\in {\mathbb {R}}, \quad 1\le k\le N \end{aligned}$$

and $\varPhi (x;\rho ,y)$ denotes the (unique) solution *h*(*x*) of the initial value problem

107 $$\begin{aligned} \left\{ \begin{aligned} \frac{d}{dx}h(x)&=-{\mathbf {M}}(h(x),x,x+y)h(x)+\sum _{j=1}^N{\mathbf {D}}(x,x+y)h(x),\\ h(0)&= \rho (y), \end{aligned} \right. \end{aligned}$$

where the initial condition

$$\begin{aligned} \rho \in C_T({\mathbb {R}}^{}_+,{\mathbb {R}}^{N}_+):=\{\rho \in C({\mathbb {R}}^{}_+,{\mathbb {R}}^{N}_+):\rho (t+T)=\rho (t)\}. \end{aligned}$$

We shall assume that the nonnegative cone $C_T({\mathbb {R}}^{}_+,{\mathbb {R}}^{N}_+)$ is equipped with the supremum norm $\Vert \rho (t)\Vert _{C([0,T])}$. It follows from the uniqueness results of Sect. 4.2 that the function $\varPhi (x;\rho ,y)$ is *T*-periodic in *y*.

A function $\rho \in C_T({\mathbb {R}}^{}_+,{\mathbb {R}}^{N}_+)$ is said to be an *upper* (resp. *lower*) solution to (106) if $\rho \ge \widetilde{{\mathcal {K}}}\rho$ (resp. $\rho \le \widetilde{{\mathcal {K}}}\rho$). It follows from Lemma 5 and condition (H4), it follows that $\widetilde{{\mathcal {K}}}$ has a bounded range:

108 $$\begin{aligned} \Vert \widetilde{{\mathcal {K}}}(\rho )\Vert _{C([0,T])}\le \omega _2. \end{aligned}$$

In particular, any solution of the characteristic Eq. (106) is bounded by $\omega _2$.

Recall that a (nonlinear) operator is called *absolutely continuous* if it is continuous and maps bounded sets into relatively compact sets.

### Lemma 15

$\widetilde{{\mathcal {K}}}:C_T({\mathbb {R}}^{}_+,{\mathbb {R}}^{N}_+)\rightarrow C_T({\mathbb {R}}^{}_+,{\mathbb {R}}^{N}_+)$ is an absolutely continuous operator.

### Proof

By the Arzela–Ascoli theorem it suffices to show that the family of functions

$$\begin{aligned} \{\widetilde{{\mathcal {K}}}\rho : \rho \in C_T({\mathbb {R}}^{}_+,{\mathbb {R}}^{N}_+) \quad \text {and}\quad \Vert \rho (t)\Vert _{C([0,T])}\le R\} \end{aligned}$$

is uniformly bounded and equicontinuous for any $R>0$. The first property is by (108). In order to prove that the family is equicontinuous, we estimate $|\widetilde{{\mathcal {K}}}\rho (t_1)-\widetilde{{\mathcal {K}}}\rho (t_2)|$ for $|t_1-t_2|<\delta _1$ and for any $\rho \in C_T({\mathbb {R}}^{}_+,{\mathbb {R}}^{N}_+)$ such that $\Vert \rho (t)\Vert _{C([0,T])}\le R$. To this end, we assume that $\tau :=t_2-t_1>0$ is such that

$$\begin{aligned} \tau<\delta _1<\frac{1}{2}\min \{a_m,b_1-A_m\}, \end{aligned}$$

where $a_m,$ $A_m$ and $b_1$ are the structure constants in (H1) and (H4) . Rewriting

$$\begin{aligned} \widetilde{{\mathcal {K}}}\rho (t_2)&=\int _{a_m}^{A_m}\mathbf{m }(a,t_2)\varPhi (a,t_2-a;\rho )da \\&=\int _{a_m-\tau }^{A_m-\tau }\mathbf{m }(a+\tau ,t_1+\tau )\varPhi (a+\tau ,t_1-a;\rho )da \end{aligned}$$

and using the property that $\mathbf{m }(a,t_i)=0$ for any *a* outside $[a_m,A_m]$ for $i=1,2$ we obtain component-wise estimates

$$\begin{aligned} |(\widetilde{{\mathcal {K}}}\rho (t_1))_k-(\widetilde{{\mathcal {K}}}\rho _k(t_2))_k|&\le \int _{a_m/2}^{A_m}|m_k(a+\tau ,t_1+\tau )-m_k(a,t_1)|\varPhi _k(a+\tau ,t_1-a;\rho )\,da\\&\quad +\int _{a_m/2}^{A_m} m_k(a,t_1)|\varPhi _k(a+\tau ,t_1-a;\rho )-\varPhi _k(a,t_1-a;\rho )|\,da\\&=:I_1+I_2 \end{aligned}$$

We have by (43) that for any $\tau >0$ and $t_1\in {\mathbb {R}}^{}$

$$\begin{aligned} \int _{a_m/2}^{A_m}\varPhi _k(a+\tau ,t_1-a;\rho )\,da\le \int _{a_m/2}^{A_m}\frac{\omega _1}{(a+\tau )^{-1/\gamma }}\,da\le \omega _1\int _{a_m/2}^{A_m}\frac{1}{a^{-1/\gamma }}\,da=: C_1 \end{aligned}$$

where $C_1$ depends only on the structural constants. Next, since $m_k(a,t)$ is a *T*-periodic in *t*, by (H4) $m_k$ is uniformly continuous on the strip $[a_m,A_m]\times {\mathbb {R}}^{}$. Since ${{\,\mathrm{\mathrm {supp}}\,}}m_k\subset [a_m,A_m]\times {\mathbb {R}}^{}$, there exists $\delta _2>0$ such that for any $1\le k\le N$, $a\in [0,A_m]$ and $|\tau |<\delta _2$ one has the inequality

$$\begin{aligned} |m_k(a+\tau ,t_1+\tau )-m_k(a,t_1)| <\frac{\epsilon }{2C_1}. \end{aligned}$$

This yields $I_1<\epsilon /2$. In order to estimate $I_2$, we notice that $\varPhi _k(x):=\varPhi _k(x,t_1-a;\rho )$ is the solution of the initial problem (107). Notice that by (42)

$$\begin{aligned} \max _{1\le k\le N}\Vert \varPhi _k\Vert _{C([0,b])}\le \sqrt{N}e^{N\Vert {\mathbf {D}}\Vert b}\Vert \rho \Vert _\infty \le C_2:=R\sqrt{N}e^{N\Vert {\mathbf {D}}\Vert b}. \end{aligned}$$

Let

$$\begin{aligned} C_3&:=\max \{M_k(v,a,t): 0\le v\le C_1, \, 0\le \frac{1}{2}(b_1+A_m), \, 0\le t\le T\}\\&:=\max \{M_k(v,a,t): 0\le v\le C_1, \, 0\le \frac{1}{2}(b_1+A_m), \, 0\le t<\infty \}, \end{aligned}$$

where the latter equality is by the periodicity. Therefore, applying the mean value theorem to (107) we obtain for any $0\le x_1<x_2<A_m+\delta _1$ and for some $\xi \in (x_1,x_2)$ that

$$\begin{aligned} \frac{|\varPhi _k(x_1)-\varPhi _k(x_2)|}{x_2-x_1} \le (|M_k(\varPhi _k(\xi ),\xi ,t_1-a)|+ N\Vert {\mathbf {D}}\Vert )C_2\le (C_3+ N\Vert {\mathbf {D}}\Vert )C_2=:C_4, \end{aligned}$$

where $C_4$ depends only on the structure conditions and *R*. This readily implies

$$\begin{aligned} I_2\le C_4A_m\Vert {\mathbf {m}}\Vert _\infty \delta _1. \end{aligned}$$

Choosing $\delta _1$ small enough, yields the desired conclusion. $\square$

### Proposition 13

For any $\rho ^+(t)$ such that $\rho ^+(t)\ge \omega _2\cdot {\mathbf {1}}_{N}$, where $\omega _2$ is defined by (51), the limit

$$\begin{aligned} \theta (t):=\lim _{i\rightarrow \infty }\widetilde{{\mathcal {K}}}^i(\rho ^+(t)) \end{aligned}$$

exists and is a solution to the characteristic Eq. (106). Furthermore, the limit $\theta (t)$ does not depend on a particular choice of $\rho ^+(t)$ and it is the maximal solution to Eq. (106) in the sense that if $\rho (t)$ is any solution to the characteristic Eq. (106) then $\rho (t)\le \theta (t)$. Furthermore, if $\rho ^-(t)$ is a lower solution then $\theta (t)\ge \rho ^-(t)$.

### Proof

Since $\widetilde{{\mathcal {K}}}\rho ^+(t)\le \omega _2 \cdot \mathbf{1 }_{N}\le \rho ^+(t)$ and by the monotonicity of $\widetilde{{\mathcal {K}}}$ we get:

$$\begin{aligned} \widetilde{{\mathcal {K}}}^{j+1}\rho (t)\equiv \widetilde{{\mathcal {K}}}^j\widetilde{{\mathcal {K}}}\rho ^+(t)\le \widetilde{{\mathcal {K}}}^j \rho ^+(t), \end{aligned}$$

which implies that $\{\rho ^{(j)}(t)\}$ is a non-increasing sequence. The sequence is bounded from below because $\widetilde{{\mathcal {K}}}^j \rho ^+\ge 0$, therefore there exists a pointwise $\lim _{j\rightarrow \infty } \widetilde{{\mathcal {K}}}^j \rho ^+(t)=:\theta (t)$. The sequence $\{\rho ^{(j)}(t)\}$ is uniformly bounded by the constant $\omega _2$. Applying Lemma 15 to family $\{\rho ^{(j)}_k(t)\}$ implies that the convergence is in fact uniform on each compact subset of ${\mathbb {R}}$. Thus $\theta$ is a nonnegative continuous *T*-periodic solution of (106). The rest of the proof is analogous to the proof of Proposition 9. $\square$

In the remaining part of this section we additionally assume that additionally condition (H6) holds. In that case, due to the periodicity, the infimum in (H6) can be replaced by the minimum. Then arguing similarly to Lemma 10, one can verify that for any $\rho (t)\in C_T({\mathbb {R}}^{}_+,{\mathbb {R}}^{N}_+)$ and $0<\lambda _1<\lambda _2$,

$$\begin{aligned} \frac{1}{\lambda _1}\widetilde{{\mathcal {K}}}(\lambda _1 \rho )\gg \frac{1}{\lambda _2}\widetilde{{\mathcal {K}}}(\lambda _2\rho ), \end{aligned}$$

hence the corresponding next generation operator is well-defined defined by

$$\begin{aligned} \widetilde{{\mathscr {R}}}_0\rho =\lim _{\lambda \rightarrow +0}\frac{1}{\lambda }\widetilde{{\mathcal {K}}}(\lambda \rho )=\int _0^{\infty }{\mathbf {m}}(a,t){\mathbf {Y}}(a; \rho ,t-a)\,da, \end{aligned}$$

where ${\mathbf {Y}}(x,y;\rho )$ is the solution of the linear system

$$\begin{aligned} \frac{d {\mathbf {Y}}(x,y;\rho )}{dx}&=({\mathbf {D}}(x,x+y)-{\mathbf {M}}(0,x,x+y)) {\mathbf {Y}}(x,x+y;\rho ), \\ {\mathbf {Y}}(0,y;\rho )&=\rho (y). \end{aligned}$$

Let ${R_0}$ denote the largest eigenvalue of $\widetilde{{\mathscr {R}}}_0$ and let $\theta =\theta (t)\in C_T({\mathbb {R}}^{}_+,{\mathbb {R}}^{N}_+)$ be the maximal solution of Eq. (106). Then the following results are established similarly to Theorems 1, 2 and 3 respectively.

### Theorem 4

If ${R_0}\le 1$, then the characteristic Eq. (106) has no nontrivial solutions (in particular, $\theta \equiv 0$). If ${R_0}>1$, then $\theta \gg 0$ is the only nontrivial solution of Eq. (106).

### Theorem 5

If ${\mathcal {F}}{\mathbf {f}}(t) \not \equiv 0$ and $\chi (t)$ is a solution to (48) then $\lim _{t\rightarrow \infty }\chi (t)=\theta (t)$.

### Theorem 6

Let ${\mathbf {P}}(t)=\int _0^{B(t)}{\mathbf {n}}(a,t)\,da$ be the total multipatch population. If ${R_0}\le 1$, then ${\mathbf {P}}(t)\rightarrow 0$ as $t\rightarrow \infty$. If ${R_0}>1$, then

$$\begin{aligned} \lim _{t\rightarrow \infty }{\mathbf {P}}(t) = \int _0^{b}\phi (a;\theta )\,da, \end{aligned}$$

where $\theta$ is the maximal solution to the characteristic Eq. (106).

## Irregularly varying environment

In order to study asymptotic behavior of the solution to the model (1)–(4) in the case when temporal variation is irregular, we assume that the vital rates, regulating function and dispersion coefficients are bounded from below and above by equiperiodic functions for large *t*. These periodic functions define two auxiliary periodic problems, whose solutions provide upper and lower bounds to a solution of the original problem. This leads us to two-side estimates of a solution to the original problem for large *t*.

More precisely, throughout this section we shall suppose that there exists $T_1\ge 0$ and *T*-periodic functions $m^{\pm }_k$, $M^{\pm }_k$ and $D^{\pm }_{kj}$ such that for any $a\ge 0$ and $t\ge T_1$

109 $$\begin{aligned} \begin{aligned} {\mathbf {m}}^-(a,t)&\le {\mathbf {m}}(a,t) \le {\mathbf {m}}^+(a,t), \\ {\mathbf {M}}^+(a,t)&\le {\mathbf {M}}(a,t) \le {\mathbf {M}}^-(a,t),\\ {\mathbf {D}}^-(a,t)&\le {\mathbf {D}}(a,t) \le {\mathbf {D}}^+(a,t). \end{aligned} \end{aligned}$$

As in Sect. 6, one can consider the corresponding characteristic equations

$$\begin{aligned} \widetilde{{\mathcal {K}}}^{\nu }\rho ^{\nu }(t)=\rho ^{\nu }(t), \quad t\in {\mathbb {R}}^{}, \end{aligned}$$

where $\nu$ denote − or $+$, and the operators $\widetilde{{\mathcal {K}}}^{\nu }$ are defined component-wise by

110 $$\begin{aligned} \widetilde{{\mathcal {K}}}^{\nu }\rho ^\nu (t):=\int _0^{\infty }{\mathbf {m}}^{\nu }(a,t) \varPhi ^{\nu }(a,t-a;\rho ^\nu )\,da, \quad t\in {\mathbb {R}}^{}_+, \quad 1\le k\le N, \end{aligned}$$

and $\varPhi ^{\nu }(x,y;\rho )$ is the unique solution of the system

$$\begin{aligned} \frac{d\varPhi ^{\nu }(x,y;\rho )}{dx}&=-{\mathbf {M}}^{\nu }(\varPhi ^{\nu }(x,y;\rho ),x,x+y) \varPhi ^{\nu }(x,y;\rho ) +{\mathbf {D}}^{\nu }(x,x+y)\varPhi ^{\nu }(x,y;\rho ), \\ \varPhi ^{\nu }(0,y;\rho )&= \rho (y), \end{aligned}$$

with $\rho \in C_T({\mathbb {R}}^{}_+,{\mathbb {R}}^{N}_+)$. Then by Proposition 6

$$\begin{aligned} \rho ^{\nu }(t)={\mathcal {K}}^{\nu }\rho ^{\nu }(t)+{\mathcal {F}}^{\nu }{\mathbf {f}}(t), \end{aligned}$$

where

111 $$\begin{aligned} \begin{aligned} {\mathcal {K}}^{\nu }\rho (t)&=\int _0^t{\mathbf {m}}^{\nu }(a,t)\varPhi ^{\nu }(a;\rho ,t-a)\,da,\\ {\mathcal {F}}^{\nu }{\mathbf {f}}(t)&=\int _t^{\infty }{\mathbf {m}}^{\nu }(a,t)\varPsi ^{\nu }(a;{\mathbf {f}},a-t)\,da. \end{aligned} \end{aligned}$$

Also let us denote by ${\mathscr {R}}_0^{\pm }$ and $R_0^\pm$ the corresponding next generation operators and basic reproduction numbers. The main result of this section states that a solution of the population problem in an irregularly changing environment can be estimated by the corresponding solutions of the associated periodically varying population problems.

### Theorem 7

Let $\chi (t)$ be a solution to Eq. (48). Then the following dichotomy holds:

(i) If $$R_0^+ \le 1$$, then $$\lim _{t\rightarrow \infty }\chi (t)=0$$.
(ii) If $R_0^- >1$ and ${\mathcal {F}}^-{\mathbf {f}}(t)\not \equiv 0$, then for any $\epsilon >0$ there exists $T_2>0$ such that
  112 $$\begin{aligned} \rho ^-(t)-\varepsilon \le \chi (t) \le \rho ^+(t)+\varepsilon \quad \forall t>T_2, \end{aligned}$$
  where $\rho ^{\pm }(t)$ are solutions to (110).

### Proof

Without loss of generality $T_1\ge B(0)$. Let $R>2\omega _2$ and let us define $\{\chi ^{(j)}(t)\}_{j\ge 0}$ and $\{\chi ^{(j)}_+(t)\}_{j\ge 0}$ iteratively for $t>T_1$ by

$$\begin{aligned}&\chi ^{(j+1)}(t)={\mathcal {K}}\chi ^{(j)}(t) + {\mathcal {F}}{\mathbf {f}}(t), \quad \chi ^{(0)}(t)=R\\&\chi ^{(j+1)}_+(t)={\mathcal {K}}^+ \chi ^{(j)}_+(t), \qquad \qquad \chi ^{(0)}_+(t)=R, \end{aligned}$$

where the operators ${\mathcal {K}}$ and ${\mathcal {F}}$ are defined by (49) and (50) respectively.

Arguing as in the proof of Proposition 7, we obtain the existence of the limit $\lim _{j\rightarrow \infty }\chi ^{(j)}(t)=\chi (t)$, where $\chi (t)$ is a solution to (48). Also by Proposition 13, $\lim _{j\rightarrow \infty }\chi ^{(j)}_+(t)=\chi ^+(t)$, where $\chi ^+(t)$ is the maximal solution to (110). We will prove by induction that for any $j\ge 0$ there holds

113 $$\begin{aligned} \chi ^{(j)}(t)\le \chi ^{(j)}_+(t), \quad \forall t>T_1+jA_m. \end{aligned}$$

For $j=0$ the claim follows from $\chi ^{(0)}(t)=\chi ^{(0)}_+(t)=R$ for $t>T_1$. Next, by our choice of $T_1$, ${\mathcal {F}}{\mathbf {f}}(t)={\mathcal {F}}^+{\mathbf {f}}(t)=0$ for $t>T_1$. Since $\varPhi (0; \chi ^{(0)},y)=\varPhi ^{\pm }(0; \chi ^{(0)}_+,y)=R$ for any $y\ge 0$ and the structure parameters are estimated by (109), one easily deduces from the definition of $\varPhi ^{\nu }(x,y;\rho )$ that $\varPhi (a; \chi ^{(0)},t-a)\le \varPhi ^{+}(a; \chi ^{(0)}_+,t-a)$ for $a\ge 0$ and $t-a\ge T_1$. Since

$$\begin{aligned} \chi ^{(1)}(t) = \int _0^t{\mathbf {m}}(a,t)\varPhi (a;\chi ^{(0)},t-a)\,da= \int _{a_m}^{A_m}{\mathbf {m}}(a,t)\varPhi (a;\chi ^{(0)},t-a)\,da \end{aligned}$$

and $t-a>T_1$ for all $a\in [a_m,A_m]$ and $t>T_1+A_m$ we obtain

$$\begin{aligned} \chi ^{(1)}(t) = \int _0^t{\mathbf {m}}(a,t)\varPhi (a;\chi ^{(0)},t-a)\,da \le \int _0^t{\mathbf {m}}^+(a,t)\varPhi ^+(a;\chi ^{(0)}_+,t-a)\,da = \chi ^{(1)}_+(t). \end{aligned}$$

This proves the induction assumption for $j=1$. Now suppose that the induction claim holds for some $j\ge 1$. Arguing similarly, we obtain for any $t>T_1+(j+1)A_m$ that

$$\begin{aligned} \chi ^{(j+1)}(t) = {\mathcal {K}}\chi ^{(j)}(t) \le {\mathcal {K}}\chi ^{(j)}_+(t) \le {\mathcal {K}}^+\chi ^{(j)}_+(t)= \chi ^{(j+1)}_+(t), \end{aligned}$$

which proves (113). Therefore, passing to the limit we obtain

114 $$\begin{aligned} \chi (t)=\lim _{j\rightarrow \infty }\chi ^{(j)}(t) \le \lim _{j\rightarrow \infty }\chi _+^{(j)}(t) =\chi ^+(t). \end{aligned}$$

If $R_0^+ \le 1$, then by Theorem 5$\lim _{t\rightarrow \infty }\chi ^+(t)=0$, hence (114) implies (i).

To proceed with (ii) notice that (114) already yields the upper estimate in (112). It remains to show that there exists a lower solution $\chi ^-(t)$ to $\chi (t)={\mathscr {L}}_{\mathbf {f}}^-\chi (t)$. We use auxiliary sequence $\{\chi ^{(j)}_-(t)\}$ given by

$$\begin{aligned} \chi ^{(j+1)}_-(t)={\mathscr {L}}_{\mathbf {f}}^-\chi ^{(j)}(t), \quad \chi ^{(0)}_-(t)=0, \end{aligned}$$

to define function

115 $$\begin{aligned} \chi ^-(t) =\left\{ \begin{array}{ll} \chi ^{(j)}_-(t), &{}\quad 0\le t\le T_1\\ \lambda \rho ^-(t), &{}\quad t>T_1, \end{array} \right. \end{aligned}$$

where $\rho ^-$ is a solution to the characteristic equation $\widetilde{{\mathcal {K}}}^-\rho ^-(t)=\rho ^-(t)$ and $\lambda >0$ is sufficiently small.

Notice first that the sequence $\{\chi ^{(j)}_-(t)\}$ is nondecreasing in *j* and that each $\chi ^{(j)}_-(t)$ satisfies $\chi ^{(j)}_-(t)\le {\mathscr {L}}_{\mathbf {f}}^-\chi ^{(j)}(t)$, i.e., it is a lower solution to equation $\chi (t)={\mathscr {L}}_{\mathbf {f}}^-\chi (t)$. Hence, $\chi ^-(t)$ defined by (115) is a lower solution in the interval $t\in [0,T_1]$ for sufficiently large *j*. Now suppose that $t\in [T_1,T_1+A_m]$. By (H1) we have ${\mathcal {F}}^-{\mathbf {f}}(t)=0$ and ${\mathscr {L}}_{\mathbf {f}}^-\chi ^-(t)={\mathcal {K}}^-\chi ^-(t)$. Thus, (110) and (115) imply that

116 $$\begin{aligned}&{\mathcal {K}}^-\chi ^-(t) - \chi ^-(t)= \int _0^{A_m}{\mathbf {m}}^-(a,t)\varPhi ^-(a;\chi _-,t-a)\,da - \lambda \rho ^-(t) \nonumber \\&\quad = \int _0^{t-T_1}{\mathbf {m}}^-(a,t)(\varPhi ^-(a;\lambda \rho ^-,t-a) -\lambda \varPhi ^-(a;\rho ^-,t-a))\,da \end{aligned}$$

117 $$\begin{aligned}&\qquad + \int _{t-T_1}^{A_m}{\mathbf {m}}(a,t)(\varPhi (a;\chi ^{(j)}_-,t-a) -\lambda \varPhi ^-(a;\rho ^-,t-a))\,da. \end{aligned}$$

Arguing similarly to the proof of Theorem 2, yields that integrals (116) and (117) are nonnegative. This proves that $\chi ^-(t)\le {\mathscr {L}}^-\chi ^-(t)$ for $t\in [T_1,T_1+A_m]$.

For $t>T_1+A_m$, we have that ${\mathcal {F}}^-{\mathbf {f}}(t)=0$, and ${\mathscr {L}}_{\mathbf {f}}^-\chi ^-(t)={\mathcal {K}}^-\chi ^-(t)$, hence

$$\begin{aligned} ({\mathcal {K}}^-\chi ^- - \chi ^-)_k(t) = \int _0^{A_m}m_k^-(a,t)(\varPhi _k^-(a;\lambda \rho ^-,t-a)-\lambda \varPhi _k^-(a;\rho ^-,t-a))\,da \ge 0. \end{aligned}$$

This proves that function $\chi ^-(t)$ defined by (115) is a lower solution of equation $\chi (t)={\mathscr {L}}^-\chi (t)$. Therefore,

118 $$\begin{aligned} \chi ^-(t) \le \chi (t), \quad t\ge 0. \end{aligned}$$

If $R_0^- >1$, then $R_0^+ >1$ and characteristic Eq. (110) have nontrivial solutions $\rho ^{\pm }(t)$. Then by virtue of Theorem 5, $\lim _{t\rightarrow \infty }\chi ^-(t) = \rho ^-(t)$ and $\lim _{t\rightarrow \infty }\chi ^+(t) = \rho ^+(t)$. Passing to the limit in (113) and (118) yields (112). $\square$

## Applications

In this section we consider two simple applications of our approach showing how dispersion promotes survival of a population on sink patches. In the usual situation, a habitat is a mixture of sources and sinks. Our first example shows that permanence on all patches is possible if the patches are connected and if emigration from sources is sufficiently small and does not cause extinction of a local subpopulation. Some researchers indicate that survival of migrating species is possible even if all occupied patches are sinks, see Jansen and Yoshimura (1998). Taking migratory birds as an example, we demonstrate that this is possible under certain conditions.

### A single source and multiple sinks

In order to demonstrate the influence of dispersion on persistence of population, we compare a system with *N* isolated patches with the corresponding system with dispersion. Recall that in the isolated case, ${\mathbf {D}}(a,t)\equiv 0$ implying by (79) that the basic reproduction number of the *k*th patch is given by

$$\begin{aligned} R_0^ {(k)}=\int _0^{\infty }m_k(a)\varPi _k(v)\,da, \end{aligned}$$

where $\varPi _k(v)=e^{-\int _0^a\mu _k(v)\,dv}$ is the survival probability.

In this case the spectrum of the next generation operator is

$$\begin{aligned} {{\,\mathrm{\mathrm {spectrum}}\,}}({\mathscr {R}}_{0})=\{R_0^ {(1)},\ldots ,R_0^ {(N)}\}. \end{aligned}$$

We assume that $R_0^ {(1)}>1$ and $R_0^ {(k)}\le 1$, for $k\ge 2$. In the biological terms, this is equivalent to saying that the first patch is a source and all other patches are sinks. Without migration, the population will persist on the first patch and become extinct on all other patches. For details about the age-structured logistic model that we used to describe isolated patches, we refer readers to Kozlov et al. (2017). Under the made assumptions,

$$\begin{aligned} \lim _{t\rightarrow \infty }\rho _1(t)&=\rho ^*_1, \\ \lim _{t\rightarrow \infty }\rho _k(t)&=0, \quad 2\le k\le N, \end{aligned}$$

where $\rho ^*_1>0$ is uniquely determined by

$$\begin{aligned} \int _0^{\infty }\frac{m_1(a)\varPi _1(v)}{1+\rho ^*_1(1-\varPi _1(v))}\,da=1. \end{aligned}$$

Now let us allow a small migration between patches and assume that there also holds $R_0^{(1)}>1$ and $R_0^ {(k)}\le 1$, for $k\ge 2$. Let us suppose that the dispersion coefficients

$$\begin{aligned} {\mathbf {D}}(a)=\varepsilon {\mathbf {B}}(a), \end{aligned}$$

where $\varepsilon >0$ is a small number and the parameters $B_{kj}(a)$ satisfy conjecture (H3). Then the standard linearization argument shows that the solution to the corresponding time-independent model

119 $$\begin{aligned} \begin{aligned} \frac{d\varphi (a;\rho )}{da}&= -{\mathbf {M}}(a)\varphi (a;\rho ) +\epsilon {\mathbf {B}}(a)\varphi (a;\rho ), \quad \varphi (0;\rho )&=\rho , \end{aligned} \end{aligned}$$

is given by

$$\begin{aligned} \varphi _k(a;\rho )=\varPi _k(v)\left( \rho _k + \varepsilon \int _0^a \sum _{j=1}^N\rho _jB_{kj}(s)\frac{\varPi _j(s)}{\varPi _k(s)}\,ds\right) + O(\varepsilon ^2). \end{aligned}$$

Therefore, the next generation operator takes the form

$$\begin{aligned} ({\mathscr {R}}_{0}\rho )_k = R_0^ {(k)}\rho _k + \varepsilon \int _0^{\infty }m_k(a)\varPi _k(v)\int _0^a \sum _{j=1}^N\rho _jB_{kj}(s)\frac{\varPi _j(s)}{\varPi _k(s)}\,ds\,da + O(\varepsilon ^2). \end{aligned}$$

Then latter relation yields

$$\begin{aligned} {\mathscr {R}}_{0}={{\,\mathrm{\mathrm {diag}}\,}}(R_0^ {(1)},\ldots ,R_0^ {(N)})+\epsilon {\mathscr {B}}+O(\varepsilon ^2) \end{aligned}$$

Now, recall that if *A* is a symmetric matrix and *x* is an eigenvector with a simple eigenvalue $\lambda$ then the corresponding perturbed eigenvalue of $A+\epsilon B$ (*B* may not be symmetric) is given by

$$\begin{aligned} \lambda +\epsilon \mu +O(\epsilon ^2), \quad \mu =x^tBx/|x|^2. \end{aligned}$$

For $\varepsilon =0$, the largest eigenvalue is $R_0^ {(1)}$ with the eigenvector $e_1=(1, 0,\ldots , 0)$. The perturbed eigenvalue, which will be the basic reproduction number for the next generation operator ${\mathscr {R}}_{0}$, is

$$\begin{aligned} R_0=R_0^ {(1)}+\varepsilon \int _0^{\infty }m_1(a)\varPi _1(v)B_{11}(a)\,da + O(\varepsilon ^2), \end{aligned}$$

thus $R_0>1$ for small enough $\varepsilon >0$ provided that tyhe function $B_{11}(a)\le 0$ everywhere and strictly negative in at least one point of the support of $m_1$. Thus shows that survival on all patches is possible if emigration from the source is sufficiently small.

### Multiple sinks, without a source

We consider an extreme situation when a population inhabits two patches and the basic reproduction number on *each* patch is less or equal to one. A realistic example for this kind of situation is a population of migratory birds. Their habitats consists of two patches: breeding range (characterized by the high birth rate in summer and high death rate in winter) and non-breeding range (low birth and death rates). Thus, the breeding range is a sink because of the winter conditions, and the non-breeding range is a sink because of too few births. We will demonstrate that, even in this case, there is a chance of survival if the structure parameters are suitably chosen.

Let the death rates $\mu _1>\mu _2>0$ be constant on the supports ${{\,\mathrm{\mathrm {supp}}\,}}m_1=[c_1,d_1]$ and ${{\,\mathrm{\mathrm {supp}}\,}}m_2=[c_2,d_2]$, respectively, where $c_i,d_i$ will be chosen later. In addition, suppose that

$$\begin{aligned} R_0^ {(k)}=\int _{c_k}^{d_k}m_k(a)e^{-\mu _ka}\,da=1, \quad k=1,2, \end{aligned}$$

This implies extinction of population on both patches if there is no dispersal. If the dispersion matrix *D* satisfies

$$\begin{aligned} D=\varepsilon B, \quad B= \left( \begin{array}{cc} -1 &{} 1 \\ 1&{} -1 \end{array} \right) , \end{aligned}$$

then the solution to the system (119) for $N=2$ is given by

$$\begin{aligned} \varphi _k(a;\rho )=e^{-\mu _k a}(\rho _k+\varepsilon h_k(a,\rho )+O(\varepsilon ^2)), \quad k=1,2, \end{aligned}$$

where

$$\begin{aligned} \left\{ \begin{array}{ccc} \frac{dh_1(a,\rho )}{da} = -\rho _1+e^{(\mu _1-\mu _2)a}\rho _2, \quad h_1(0)=0, \\ \frac{dh_2(a,\rho )}{da} =-\rho _2+e^{(\mu _2-\mu _1)a}\rho _1, \quad h_2(0)=0. \end{array} \right. \end{aligned}$$

A solution to this system is given by

$$\begin{aligned} \left\{ \begin{array}{ccc} h_1(a,\rho )=-\rho _1a+\frac{1}{\mu _1-\mu _2}(e^{(\mu _1-\mu _2)a}-1)\rho _2, \\ h_2(a,\rho )=-\rho _2a+\frac{1}{\mu _2-\mu _1}(e^{(\mu _2-\mu _1)a}-1)\rho _1. \end{array} \right. \end{aligned}$$

Then, the next generation operator satisfies

$$\begin{aligned} ({\mathscr {R}}_{0}\rho )_k = \rho _k + \varepsilon \int _{c_k}^{d_k}m_k(a)e^{-\mu _ka}h_k(a,\rho )\,da + O(\varepsilon ^2), \quad k=1,2. \end{aligned}$$

In the matrix form this becomes

120 $$\begin{aligned} {\mathscr {R}}_0\rho = \rho +\varepsilon {\mathscr {P}}\rho +O(\varepsilon ^2\rho ), \end{aligned}$$

where

$$\begin{aligned} {\mathscr {P}}=\left( \begin{array}{ll} -1 &{}\quad \displaystyle \int _{c_1}^{d_1}\frac{m_1(a)e^{-\mu _1a}(e^{(\mu _1-\mu _2)a}-1)}{\mu _1-\mu _2}\,da \\ \displaystyle \int _{c_2}^{d_2} \frac{m_2(a)e^{-\mu _2a}(e^{(\mu _2-\mu _1)a}-1)}{\mu _2-\mu _1}\,da &{}\quad -1 \end{array}\right) . \end{aligned}$$

Thus, to show that $R_0>1$, it is sufficient to show that ${\mathscr {P}}\rho >0$ for some choice of parameters and certain vector $\rho$.

Using

$$\begin{aligned} \psi (z)=z^{-2}(e^z-1-z)=\frac{1}{2}+\frac{z}{3!}+\frac{z^2}{4!}+\cdots \end{aligned}$$

it follows that the functions $h_1$ and $h_2$ can be written as:

$$\begin{aligned} \left\{ \begin{array}{ccc} h_1(a,\rho )=(\rho _2-\rho _1)a+a^2(\mu _1-\mu _2)\psi ((\mu _1-\mu _2)a)\rho _2, \\ h_2(a,\rho )=(\rho _1-\rho _2)a+a^2(\mu _2-\mu _1)\psi ((\mu _2-\mu _1)a)\rho _1. \end{array} \right. \end{aligned}$$

Sine the function $z\psi (z)$ monotonically increases from 0 to $\infty$, there exists a unique $c^*$ such that $c^*(\mu _1-\mu _2)\psi ((\mu _1-\mu _2)c^*)=1$. Suppose that $d_2<c^*<c_1$. Let us choose parameters $\rho _1>\rho _2>0$ such that $h_1(a,\rho )>0$ for $a>c_1$ and $h_2(a,\rho )>0$ for $a<d_2$, that is

$$\begin{aligned} \left\{ \begin{array}{ccc} \rho _1-\rho _2< a(\mu _1-\mu _2)\psi ((\mu _1-\mu _2)a)\rho _2, \quad \text{ for }\; a>c_1, \\ \rho _1-\rho _2 > a(\mu _1-\mu _2)\psi ((\mu _2-\mu _1)a)\rho _1, \quad \text{ for }\; a<d_2, \end{array} \right. \end{aligned}$$

or equivalently,

$$\begin{aligned} \left\{ \begin{array}{ccc} \frac{\rho _1}{\rho _2}-1< a(\mu _1-\mu _2)\psi ((\mu _1-\mu _2)a), \quad \text{ for }\; a>c_1,\\ 1-\frac{\rho _2}{\rho _1}> a(\mu _1-\mu _2)\psi ((\mu _2-\mu _1)a), \quad \text{ for }\; a<d_2. \end{array} \right. \end{aligned}$$

We put $\rho _1=1$ and choose $\rho _2<\frac{1}{2}$ and $c_1$ and $d_2$ as solutions to equations:

$$\begin{aligned} \frac{1}{\rho _2}- 1 = c_1(\mu _1-\mu _2)\psi ((\mu _1-\mu _2)c_1) \end{aligned}$$

and

$$\begin{aligned} 1-\rho _2 = d_2(\mu _1-\mu _2)\psi ((\mu _2-\mu _1)d_2). \end{aligned}$$

It follows that ${\mathscr {P}}\rho >0$ and hence ${\mathscr {R}}_0\rho >\rho$. The latter implies that that $R_0>1$, thus ${\mathscr {R}}_0$ has an eigenvalue greater than one, which proves the permanence of population on both patches.

## Discussion

Our work advances understanding of dynamics of age-structured, density-dependent populations in patchy, temporally-variable habitats. Intra-specific competition can act in different ways and have a whole spectrum of different forms, where the pure intra-specific competition and unstructured population-wide competition are on the opposite ends. Population growth models are often based on the assumption that competition is unstructured. In contrast to this, we focus on pure intra-cohort competition as we find it more relevant for exploring ecological questions related to, for example, ontogenetic shifts.

Our results show that the use of the basic reproduction number $R_0$ for determining permanence criteria for age structured populations can be expanded from a single-patch models into multi-patch models. The main result, Theorem A (the Net Reproductive Rate Dichotomy), is applicable if such populations are regulated by density dependent mortality and live in temporally unchanging habitats or if they face periodic environmental variation as well as fluctuations of large amplitudes. This opens up the opportunity to analyse a range of possible outcomes given specific settings relevant for ecological understanding or given specific parameter sets that define the ecological question.

We use the results to further explore the dynamics of a population that inhabits both source and sinks patches. We also study if a metapopulation can persist if all patches are sinks. Our results for the first question show that permanence is possible if all patches are connected and emigration from source patches is sufficiently small and does not cause extinction of a local subpopulation. Analysis of the second question shows that permanence of a population on pure sink patches is possible for certain patterns of dispersion.

The methodology used in the proofs, for example the storng monotonicity of lower and upper bounds, is possible to expand into other areas of population dynamics. We believe that it can be applied in analysis of multispecies dynamics, such as predator-prey, or epidemiological dynamics under assumption that model parameters are time-dependent.

## Footnotes

1 In fact, with some minor modifications, all the main results remain true under a weaker assumption that the structural coefficients are rather $L^{\infty }$-functions.
